# A common genetic variation of melanoma inhibitory activity-2 labels a subtype of pancreatic adenocarcinoma with high endoplasmic reticulum stress levels

**DOI:** 10.1038/srep08109

**Published:** 2015-02-06

**Authors:** Bo Kong, Weiwei Wu, Nataliya Valkovska, Carsten Jäger, Xin Hong, Ulrich Nitsche, Helmut Friess, Irene Esposito, Mert Erkan, Jörg Kleeff, Christoph W. Michalski

**Affiliations:** 1 Department of Surgery, Technische Universität München, Munich, Germany; 2 Institute of Pathology, Technische Universität München, Munich, Germany; 3 Department of Surgery, Koc School of Medicine, Istanbul, Turkey; 4 Department of Surgery, University of Heidelberg, Germany

## Abstract

HNF1 homeobox A (HNF1A)-mediated gene expression constitutes an essential component of the secretory pathway in the exocrine pancreas. Melanoma inhibitory activity 2 (MIA2), a protein facilitating protein secretion, is an HNF1A target. Protein secretion is precisely coordinated by the endoplasmic reticulum (ER) stress/unfolded protein response (UPR) system. Here, we demonstrate that HNFA and MIA2 are expressed in a subset of human PDAC tissues and that HNF1A induced MIA2 in vitro. We identified a common germline variant of MIA2 (c.A617G: p.I141M) associated with a secretory defect of the MIA2 protein in PDAC cells. Patients carrying MIA2^I141M^ survived longer after tumor resection but the survival benefit was restricted to those patients who received adjuvant chemotherapy. The MIA2^I141M^ variant was associated with high expression of ER stress/UPR genes – in particular those of the ERN1/XBP arm – in human PDAC samples. Accordingly, PDAC cell lines expressing the MIA2^I141M^ variant expressed high levels of ERN1 and were more sensitive to gemcitabine. These findings define an interaction between the common MIA2^I141M ^variant and the ER stress/UPR system and specify a subgroup of PDAC patients who are more likely to benefit from adjuvant chemotherapy.

Pancreatic ductal adenocarcinoma (PDAC) is an extremely aggressive cancer with not fully understood disease causes^1^,^2^. Though previous genome-wide association (GWAS) and epidemiological studies have demonstrated the relevance of a set of genetic and environmental factors in the etiology of this diseas^3^,^4^,^5^, the exact mechanisms remain largely elusive. A recent genome-wide pleiotropy scan and transcriptome analysis has identified the HNF1 homeobox A (HNF1A) gene as an important player in the development of pancreatic cancer^6^,^7^, whereas the closely related gene HNF1B had no such effect^8^. HNF1A is a critical transcription factor whose mutations have previously been shown to be responsible for an autosomal dominant form of non-insulin-dependent diabetes mellitus, the maturity-onset diabetes of the young 3 (MODY3)^9^. Though Hnf1a-deficient mice developed pancreatic islets without conspicuous defects in either the β cell mass or insulin content, they displayed a compromised insulin secretion upon glucose and arginine stimulation^10^. These data suggest that HNF1A-mediated gene expression might constitute an essential component of the secretory pathway in pancreatic β cells. Similarly, pancreatic acini isolated from Hnf1a-deficient mice show a significantly impaired amylase release upon treatment with caerulein (an analog of the potent pancreatic secretagogue cholecystokinin) compared to wild-type mice^11^. These data collectively demonstrate that HNF1A-mediated gene expression constitutes an essential component of the secretory pathway in both the endocrine and the exocrine pancreas. One of the HNF1A target molecules is the melanoma inhibitory activity 2 (MIA2)^12^,^13^,^14^ which belongs to the MIA family of genes, consisting of MIA, MIA2, MIA3/Tango and otoraplin (OTOR). This is a family of secreted proteins that contain a Src-homologous SH3 structure in the N terminus. In comparison to MIA and OTOR, MIA2 and MIA3 contain a long additional peptide sequence in the C-terminus; in this regard, evidence for the involvement of MIA2 and MIA3 in protein secretion is beginning to emerge^14^,^15^,^16^,^17^. In addition, genetic variations in members of the MIA family other than MIA2 have been repetitively found to be associated with various human diseases^18^,^19^,^20^,^21^.

The exocrine pancreas is a secretory organ producing a huge amount of digestive enzymes. To fulfill this task, pancreatic acinar cells have evolved to possess an extensive endoplasmic reticulum (ER) network in which the protein synthesis/process machinery is controlled by concerted activities of the ER-assisted folding (ERAF), ER-associated degradation (ERAD) and COPII export pathways^22^,^23^,^24^. Importantly, ERAF, ERAD and COPII export are coordinated by a regulatory machinery in the ER, the unfolded-folded protein response (UPR) which serves to sense mis-folded or overloaded proteins in the ER (“ER stress”). Thereafter, the UPR activates a series of molecular events with the aim of either mitigating ER stress or inducing cell death whenever the stress is irresolvable^25^. The importance of the UPR in maintaining homeostasis of the exocrine pancreas is reflected by recent findings that genetic ablation of almost any component of the UPR in mice (e.g. ERN1 (endoplasmic reticulum to nucleus signaling 1), XBP1 (x-box binding protein 1) or PERK (PRKR-like endoplasmic reticulum kinase)) invariably results in unrecoverable ER stress that ultimately leads to acinar cell death^26^,^27^,^28^,^29^,^30^. Furthermore, the ERN1/XBP1 arm is indispensable for embryonic development of the exocrine pancreas (likely through a crosstalk with developmental pathways) and promotes cell survival upon acinar cell damage^28^,^31^.

In addition, recent reports have demonstrated the relevance of the HNF1/MIA2 axis in hepatocellular carcinogenesis (HCC)^13^. However, it remains unknown how a secretory axis of the pancreas might influence pancreatic ductal carcinogenesis - a malignancy that likely originates from the transformation of (oncogenesis-susceptible) cells in the exocrine pancreas^2^. Since an exocrine-like subtype of PDAC has recently been identified and because HNF1A has been shown to be a novel cancer risk gene of PDAC^32^, it is likely that the HNFA/MIA2 secretory axis is active in PDACs. However, it is unclear how pancreatic cancer cells co-opt these secretory pathways to promote carcinogenesis. Furthermore, it is unknown whether and how these secretory pathways crosstalk with the ER stress/UPR system to modify PDAC tumor biology.

## Results

### Forced HNF1A expression induces MIA2

**Figure 1:**
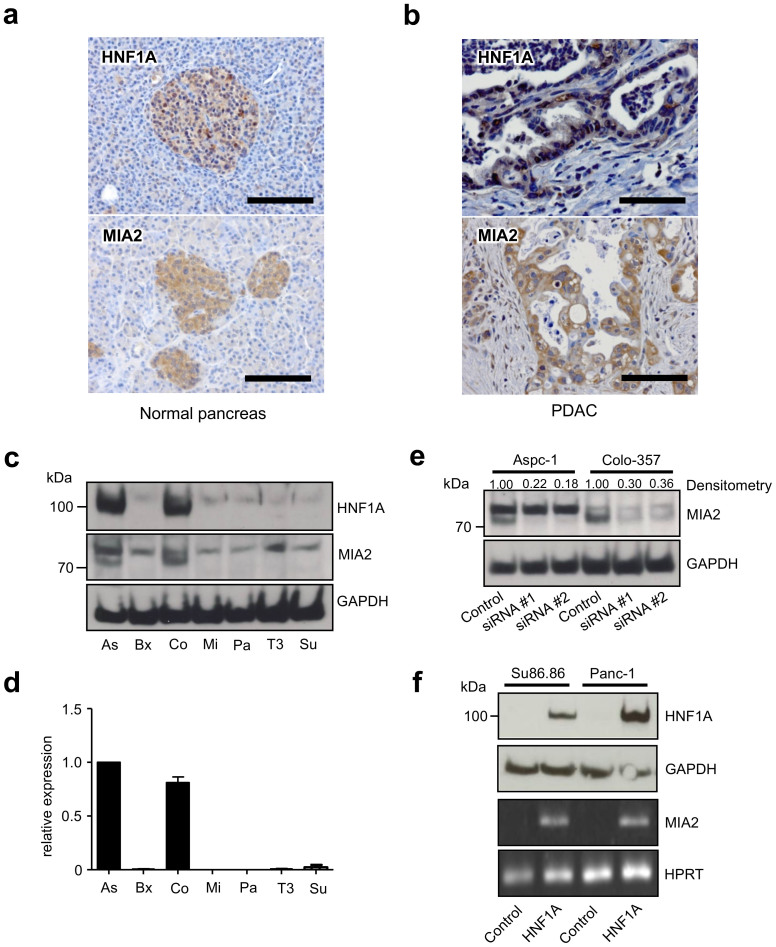
HNF1A controls MIA2 expression in PDAC. (a), Pancreatic islet cells are immunopositive for HNF1A and MIA2 (scale bar: 100 μm). (b), 73% and 52% of the samples are positive for HNF1A and MIA2, respectively (scale bar: 100 μm). (c), Protein expression of HNF1A and MIA2 in pancreatic cancer cell lines. Out of 7 tested cell lines (Aspc-1 (As), Bxpc-3 (Bx), Colo357 (Co), Mia-PaCa2 (Mi), Panc-1 (Pa), T3M4 (T3), Su86.86 (Su)), Aspc-1 and Colo-357 express HNF1A and MIA2; endogenous MIA2 is mainly detected at a size of 70 kDa (loading control: β-actin). (d), MRNA expression of MIA2 in 7 pancreatic cancer cell lines. (e), MIA2 silencing with two sets of specific siRNAs (#1 and #2) or negative control siRNA (control) at 72 h in Aspc-1 cells and Colo-357 cells; results of densitometry are shown on top of the blot. One representative blot out of three independent experiments is shown. (f), Transient expression of HNF1A in Su86.86 and Panc-1 cells by transfection of an HNF1A expression vector (upper panel), loading control: GAPDH; MIA2 mRNA expression is induced by transient expression of HNF1A in Su86.86 and Panc-1 cells. HPRT1: housekeeping gene. One of three independent experiments is shown. The gels were run under the same experimental conditions and cropped blots/gels are presented (full-length blots/gels are provided in suppl. Fig. 6 with indicated cropping lines).

Immunohistochemistry studies of HNF1A and MIA2 in the normal pancreas revealed immunoreactivity for both proteins in islets, consistent with the known HNF1A network function (Fig. 1a)^9^; however, normal pancreatic exocrine cells were generally devoid of HNF1A and MIA2 immunostaining. 73% and 52% of PDACs were immuno-positive for HNF1A (29/40) and MIA2 (32/61, Fig. 1b), respectively, which is in contrast to the MIA2 function as a major tumor suppressor in HCC^13^; this, however, is consistent with the phenotype of Hnf1a-deficient mice where growth and oncogenesis of pancreatic β cells were impaired and proliferation of hepatocytes was increased^33^. We then tested HNF1A and MIA2 expression in seven pancreatic cancer cell lines, out of which two (Aspc-1 and Colo-357) expressed both the HNF1A and MIA2 protein (Fig. 1c). MIA2 protein (at around 70 kDa) was found to be strongly expressed in Aspc-1 and Colo-357 cells, consistent with its respective mRNA expression (Fig. 1d). The specificity of the antibody was further confirmed by an siRNA assay: MIA2-specific siRNA transfection led to a decline in the intensity of the 70 kDa band (64%–82% reduction, according to densitometry), whereas no changes were observed in the presumed unspecific bands, (Fig. 1e). In line with these observations, transient expression of HNF1A in MIA2-negative cell lines (Panc-1 and Su86.86) induced MIA2 mRNA expression; however, MIA2 protein levels were below the level of detection using immunoblot analysis (Fig. 1f). Transient expression of HNF1B had no such effect (suppl. Fig. 1a and 1b), underscoring the specific role of HNF1A in controlling MIA2 expression.

### A common variation of the MIA2 gene is associated with the response to adjuvant chemotherapy

**Figure 2:**
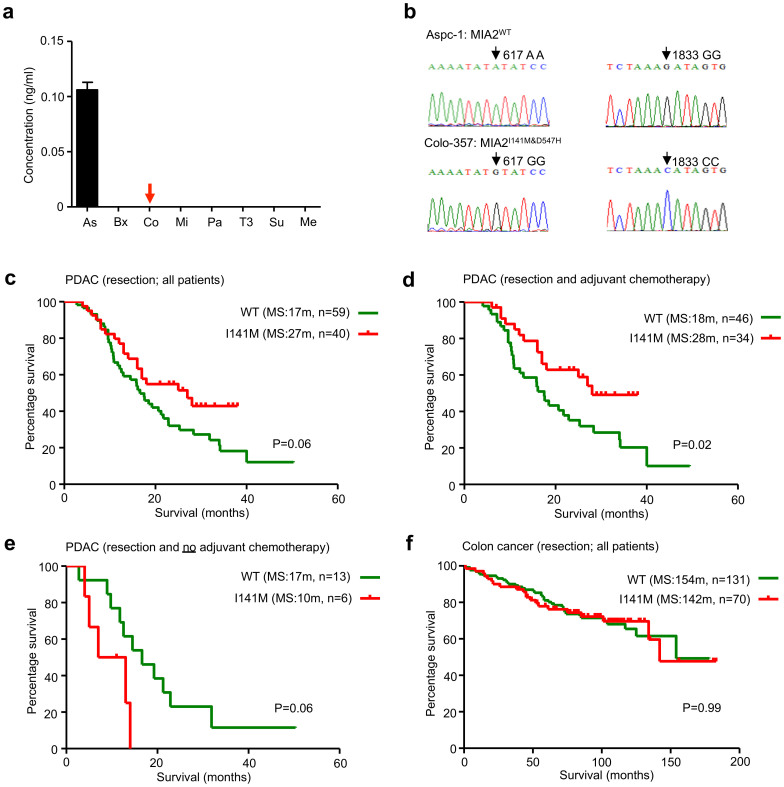
MIA2^I141M^ is associated with cancer cell secretory defects and correlates with survival of PDAC patients. (a), MIA2 levels in the supernatants of pancreatic cancer cell lines (Aspc-1 (As), Bxpc-3 (Bx), Colo357 (Co), Mia-PaCa2 (Mi), Panc-1 (Pa), T3M4 (T3), Su86.86 (Su)), as determined by ELISA. Aspc-1 cells show detectable levels of MIA2, whereas it is below the detection limit in Colo-357 cells. (b), Sequencing reveals that Colo-357 cells carry two common MIA2 variants: rs11845046 and rs10134365. (c), PDAC patients carrying MIA2^I141M^ live longer than those with MIA2^WT^ (MIA2^WT^ vs. MIA2^I141M^, median survival (MS) 27 vs. 17 months, log-rank test: p = 0.06). (d), MIA2^I141M^ carriers who received chemotherapy had a significantly longer postoperative survival (MIA2^I141M^ vs. MIA2^WT^, median survival (MS) 28 vs. 18 months, log-rank test, p = 0.02). (e), If no chemotherapy was given, MIA2^I141M^ carriers had a shorter postoperative survival (MIA2^WT^ vs. MIA2^I141M^, median survival (MS) 10 vs. 17 months, log-rank test: p = 0.06). (f), MIA2^I141M^ did not correlate with survival in colorectal cancer patients (UICC stage II, MIA2^WT^ vs. MIA2^I141M^, median survival (MS) 154 vs. 142 months, log-rank test: p = 0.99). m = months.

Since MIA2 is a secreted protein and is itself involved in protein secretion, MIA2 levels were analyzed in cancer cell supernatants by ELISA. MIA2 was detected in the supernatants of Aspc-1 cells but not in the supernatants of Colo-357 cells (Fig. 2a), suggesting a defect in MIA2 secretion in Colo-357 cells. To address the reasons for this hypothesized defect, we sequenced the MIA2 gene in the cell lines. Here, Colo-357 cells carried two common homozygous variants with the coding DNA sequences c.A617G (rs11845046) and c.G1833C (rs10134365), leading to p.I141M and p.D547H, respectively (Fig. 2b). In an attempt to interrogate the clinical relevance of these two “secretion-associated” polymorphisms, MIA2 genotypes were determined using high-resolution melting curve analyses (HRM) of tissue samples of 628 subjects. These consisted of pancreatic organ donors (26), chronic pancreatitis patients (CP, 18), pancreatic neuroendocrine tumor patients (PNTs, 38), PDAC patients (277), colorectal (230) and esophageal cancer patients (39; all shown in Table S1 and suppl. Fig. 2a, 2b, 2c). Notably, the occurrence of the MIA2^I141M^ variant strictly correlated with that of the MIA2^D547H^ variant in the first 262 tested samples, demonstrating that they are located within the same linkage disequilibrium (LD) block of the human genome. Therefore, for the other samples, only the MIA2^I141M^ variant was genotyped. As shown in Table S1, the overall frequency of the MIA2^I141M^ variant was 0.36 (ranging from 0.28 to 0.50; 628 samples) and its frequency in PDAC samples was 0.35. For 99 out of 277 PDAC samples, detailed clinical and pathological data were available (Table S2). Although the distribution of MIA2^I141M^ did not correlate with clinical parameters such as the tumor (T) stage and the grading (Table S2), Kaplan-Meier analysis revealed that PDAC patients with the MIA2^I141M^ variant tended to live longer after surgical resection than patients with MIA2^WT^ (median survival: 27 vs. 17 months, p = 0.06, Fig. 2c). Notably, further analysis revealed that the survival benefit for MIA2^I141M^ patients was predominantly derived from patients who received adjuvant chemotherapy (median survival: 28 vs. 18 months, p = 0.02, Fig. 2d) because in the no-chemotherapy group, patients with the MIA2^I141M^ variant survived relatively shorter after surgical resection (median survival: 10 vs. 17 months, p = 0.06, Fig. 2e). This suggests that the MIA2^I141M^ variant carriers are more likely to benefit from chemotherapy. However, the MIA2^I141M^ variant was not associated with survival in colon cancer (p = 0.99, Fig. 2f) or with the response to neoadjuvant chemotherapy in esophageal adenocarcinoma (data not shown).

### Expression of MIA2^I141M^ increases chemosensitivity to gemcitabine

**Figure 3:**
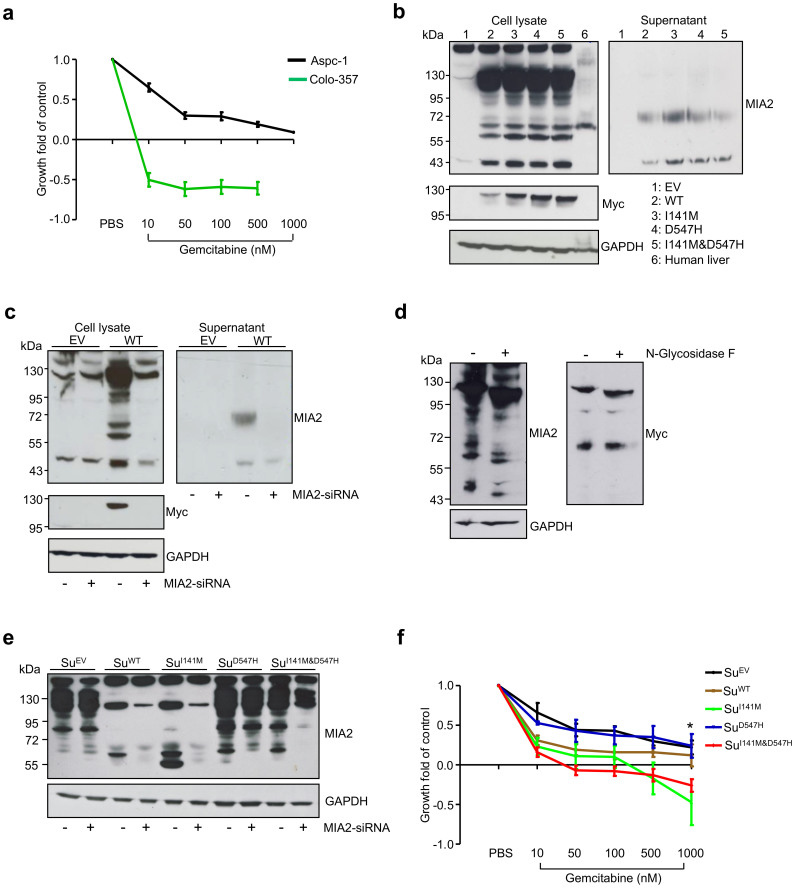
MIA2^I141M^ increases sensitivity of PDAC cells to gemcitabine. (a), Colo-357 cells are highly sensitive towards gemcitabine. Data from three independent experiments are expressed as mean ± SD. (b), Transient expression of the MIA2^WT^ and MIA2^I141M^ variants in HEK293 cells gave rise to bands at 120, 100, 70, 56 and 43 kDa (Western-blot analysis of cell lysates) while the secreted forms localized mainly at 43 and 70 kDa when the MIA2 antibody (recognizing the N-terminus of the protein) was used; interestingly, the myc-tag antibody (recognizing the C-terminus) only detected the bands at 120 kDa. (c), These bands at various sizes disappeared upon co-transfection with MIA2-specific siRNA. (d), N-Glycosidase F treatment of cell lysates from MIA2^WT^-transfected HEK293 cells partially reduced the size of the MIA2 proteins. One representative blot out of two independent experiments is shown. (e), Su86.86 cells transfected with different MIA2 expression vectors showed stable and comparable levels of MIA2; the empty vector (EV) was transfected as a control. Loading control: GAPDH; exogenous MIA2 was mainly detected at 120, 61 and 56 kDa. (f), Introduction of MIA2^I141M^ (but not of MIA2^WT^ or MIA2^D547H^) significantly increased sensitivity towards gemcitabine compared to empty vector (EV)-transfected cells in vitro, *: MIA2^I141M^ vs. MIA2^EV^: p = 0.0015; MIA2^I141M&D547H^ vs. MIA2^EV^: p = 0.0002, unpaired t-test. Data from three independent experiments are presented as mean ± SD. The gels were run under the same experimental conditions and cropped blots/gels are presented (full-length blots/gels are provided in suppl. Fig. 6 with indicated cropping lines).

Since these data in PDAC strongly suggested that the secretion-associated MIA2 polymorphisms were associated with the clinical response to adjuvant chemotherapy, we sought to determine whether this was reflected in pancreatic cancer cell lines in vitro. Indeed, a chemotherapy assay revealed that Colo-357 cells were extremely sensitive to gemcitabine treatment at a low drug dose (10 nM), which in contrast only had a marginal effect on the growth of Aspc-1 cells (Fig. 3a). Because these two cell lines are also disparate in various aspects other than the MIA2 genotypes, it is difficult to directly evaluate the biological effect of the MIA2 variants to the chemoresponse. To this end, expression vectors for wild-type MIA2 (WT), MIA2 containing either of the polymorphisms (I141M, D547H) or both (I141M & D547H) were constructed to generate pancreatic cancer cell lines expressing WT or MIA2 variants on a similar background. A myc tag was fused to the C-terminus of the exogenous MIA2 protein. To test these expression vectors, HEK293 cells were transiently transfected with the different polymorphism-containing MIA2 vectors. Interestingly, using an antibody recognizing the N-terminal region of MIA2, 5 bands shifting at the size of around 120, 100, 70, 56 and 43 kDa were detected in the HEK293 cell lysates (after transfection) whereas the endogenous MIA2 from human liver lysates was mainly detected at 70 kDa^12^,^13^, as previously described (Fig. 3b). The secreted MIA2 in the supernatants was mainly detected at a size of 43 and 70 kDa (Fig. 3b). However, no difference in the secretion of the MIA2 variants was found, most likely because of non-physiological expression of MIA2 (driven by the CMV promoter). The myc-tag antibody only recognized the 120 kDa band and no signals in the supernatants (Fig. 3b). In order to confirm that the detected bands were truly produced due to MIA2-transfection, specific MIA2 siRNAs were co-transfected with the WT-MIA2 vector. These experiments demonstrated that the bands seen at unexpected sizes disappeared following siRNA silencing of MIA2. This data indicates that these different MIA2 proteins were indeed translated from the MIA2 mRNA (Fig. 3c), suggesting that MIA2 undergoes intensive intracellular protein modifications. In this regard, two N-linked glycosylation sites at position 59 (NFT) and 367 (NDS) which are unique features of secretory proteins were found by scanning the protein sequence of MIA2^24^. A deglycosylation assay demonstrated that treating cell lysates from WT-transfected HEK293 cells with N-Glycosidase F partially lowered the size of the MIA2 proteins (Fig. 3d). These data provide evidence that MIA2 is a secretory protein that is glycosylated in the ER compartment, which is in accordance with its previously described cellular localization^14^.

Thereafter, Su86.86 cells (low MIA2 expression levels) were transfected with MIA2 or empty vectors (EV), followed by antibiotics selection. Clones expressing different forms of MIA2 at a similar level (as determined by Western-blot analysis, Fig. 3e) or carrying an empty vector were generated and termed as Su^EV^ (empty vector), Su^WT^, Su^I141M^, Su^D547H^ and Su^I141M&D547H^. These were then subjected to further functional characterization. Additionally, cDNAs from these corresponding clones were sequenced to confirm successful introduction of the MIA2 polymorphisms (suppl. Fig. 3). Compellingly and in accordance with the results of the chemosensitivity assays, expression of the MIA2^I141M^ variant specifically rendered pancreatic cancer cells more susceptible to the effects of gemcitabine (especially at high doses (1000 nM): MIA2^I141M^ vs. MIA2^EV^: p = 0.0015; MIA2^I141M&D547H^ vs. MIA2^EV^: p = 0.0002, unpaired t-test) whereas the expression of the WT or the D547H variant alone had no significant effect (Fig. 3f).

### The MIA2-ERN1 axis determines chemoresponsiveness in pancreatic cancer cells

**Figure 4:**
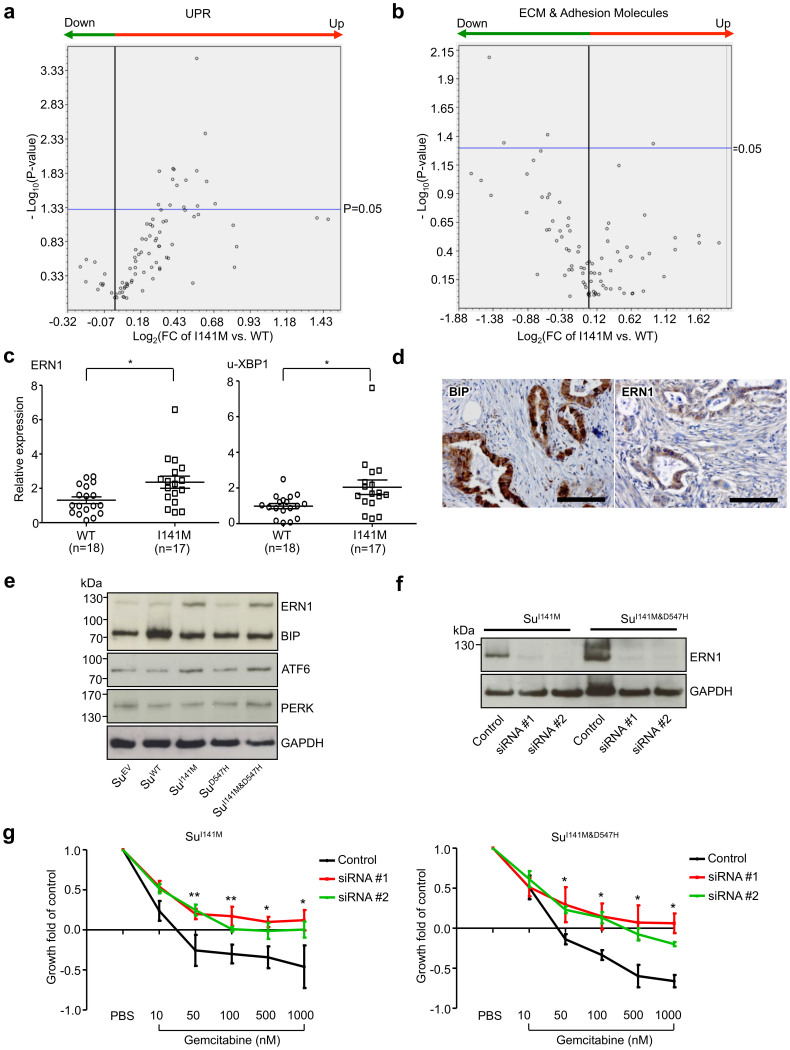
MIA2^I141M^ is associated with increased expression of ERN1/XBP1 in PDAC tissues. (a), A real-time PCR-based array was performed on 23 PDAC cancer tissues (11 MIA2^WT^ and 12 MIA2^I141M^). Compared to MIA2^WT^ samples, 18% (15/84) of UPR genes were significantly up-regulated in MIA2^I141M^ tissues. (b), However, only four ECM and adhesion molecules (ITGA2, LAMA1, MMP12 and ICAM1) were differentially expressed. (c), Up-regulation of ERN1 and the un-spliced XBP1 isoform (u-XBP1) was confirmed by QRT-PCR. Data are presented as relative expression (normalized to the median expression of ERN1 and u-XBP1 in MIA2^WT^ samples); *: p < 0.05. (d), 64% (39/61) and 35% (21/60) of the PDAC samples are immune-positive for the ER stress markers BIP and ERN1, respectively (scale bar: 100 μm). (e), MIA2^I141M^ pancreatic cancer cell lines (Su^I141M^, Su^I141M&D547H^) also expressed ERN1 at higher levels than the other cancer cell lines. No such tendency was seen for the other tested molecules (BIP, ATF6 and PERK). (f), ERN1 silencing with two sets of specific siRNAs (#1 and #2) or negative control siRNA (control) at 72 h in Su^I141M^ and Su^I141M&D547H ^cells. One representative blot out of three independent experiments is shown. (g), ERN1 silencing in Su^I141M^ and Su^I141M&D547H^ cells reverses the chemosensitive phenotype caused by expression of the MIA2^I141M^ variant. *: both #1 and #2 are significant. **: only #1 is significant, p < 0.05. Data from three independent experiments are expressed as mean ± SD. The gels were run under the same experimental conditions and cropped blots/gels are presented (full-length blots/gels are provided in suppl. Fig. 6 with indicated cropping lines).

Since MIA2 is functionally related to protein secretion and because the UPR, activated by ER stress, couples the secretory network to cell survival in the adult pancreas^26^,^27^,^28^,^29^,^30^, we hypothesized that the MIA2^I141M^ variant may influence the UPR “homeostasis” in pancreatic cancer cells. To evaluate this hypothesis, a real-time PCR-based array of a large number of genes belonging to the UPR network was performed on PDAC samples from patients with the MIA2^I141M^ variant and the MIA2^WT^. Because MIA3 (but, not yet, MIA2) has been shown to be involved in the secretion of extracellular matrix proteins (ECM), ECM and adhesion molecules were also analyzed using a PCR array^15^,^16^. In total, 23 samples were analyzed; of which 11 were MIA2 wild type and 12 carried the variants (heterozygous (9), homozygous (3)). As shown in the volcano plot (Fig. 4a), many genes involved in UPR were up-regulated in cancer tissues from MIA2^I141M^ variant carriers; 18% (15/84) of these were found to be statistically significant (p < 0.05, Table S3). In the ECM/adhesion molecule arrays, expression of only 4 genes (ITGA2, LAMA1, MMP12 and ICAM1) was significantly different and three of these were down-regulated (Fig. 4b; Table S4). Then, genes with a fold change (FC) of more than 50% were plotted (suppl. Figure 4a; Table S3). Here, of the three UPR arms (ERN1/XBP1, PERK/p-eIF2α and ATF6/nATF6α)^25^, the ERN1/XBP1 arm seemed to be particularly affected by the respective MIA2 genotype because ERN1 and XBP1 were significantly up-regulated in the MIA2^I141M^ variant carriers (suppl. Fig. 4a). Changes in ERN1 and in un-spliced XBP1 (u-XBP1) expression were also confirmed on more PDAC samples (Fig. 4c); no differences in the expression of spliced XBP1 were seen (s-XBP1, suppl. Fig. 4b, 4c). Immunohistochemical analyses revealed that 64% (39/61) and 41% (25/61) of PDAC sections were positive for BIP, a general ER stress marker and ERN1, respectively (Fig. 4d). Other UPR markers such as PERK, ATF6, PDI and Calnexin were also frequently expressed by PDAC cells (suppl. Fig. 5a and 5b). These data underscore the biological significance of ER stress and the associated UPR in pancreatic cancer. Consistently, pancreatic cancer cell lines expressing the MIA2^I141M^ variant had a higher expression of ERN1 while no such effect was found for the other tested UPR molecules (Fig. 4e). Notably, ERN1 silencing reversed the chemo-sensitive phenotype of these two cell lines (Fig. 4f, 4g), providing a molecular link between the MIA2^I141M^ variant and pancreatic cancer cell chemo-response. Due to unknown reasons, ERN1 silencing using siRNA transfection was not possible in Colo-357 cells (with endogenous MIA2^I141M^ expression). Thus, no chemotherapy study was performed in these cells.

## Discussion

**Figure 5:**
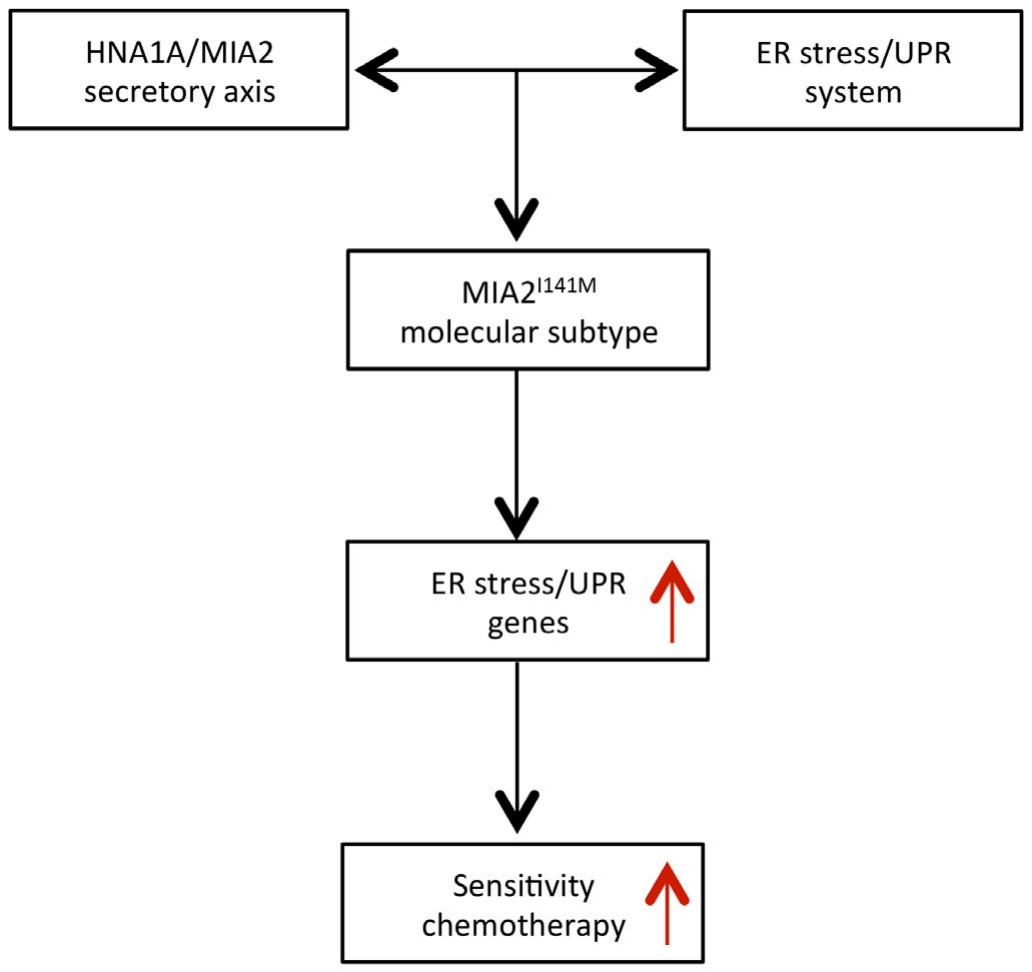
Schematic illustration shows that MIA2^I141M^ specifies a molecular subtype of chemosensitive PDAC.

Taken together, we identified a common germline variant of MIA2^I141M^ that is associated with a secretory defect of the MIA2 protein in pancreatic cancer cells. This variant was found to be related with high expression of ER stress/UPR genes in human PDAC samples. While patients carrying the MIA2^I141M^ variant tended to have a more aggressive cancer phenotype, they were more likely to respond to adjuvant chemotherapy (Fig. 5).

### Common genetic variations and pancreatic cancer biology

The completion of the Human Genome and the International HapMap Projects, together with rapid improvements in genotyping technologies, has rendered GWAS in many disease entities possible. Such studies tend to identify common (population frequencies of more than 5%) and low-risk variants (odds ratios of 1.2 to 5.0) for a given disease^34^. Recently published GWAS studies have identified a number of loci associated with susceptibility to pancreatic cancer in different populations^3^,^4^,^35^. However, in the absence of selective evolutionary pressure, these common variants only cause slight alterations of a gene's expression or a protein's function^36^,^37^. Therefore, it has been controversially discussed whether such minor effects add up to an increased cancer risk or whether rather the individual and rare mutations with large effects, which are usually below the detection range of GWAS studies, are responsible for the risk increase. Indeed, recent deep sequencing efforts of drug target genes revealed that many human rare variants potentially also influence human disease risk^38^. Nevertheless, genes with common mutations can actually frequently be mapped to core signaling pathways that operate in established pancreatic cancers^39^, which would suggest that common variants affecting cancer susceptibility may also influence the biological behavior of pancreatic cancer cells.

### The HNF1A/MIA2 axis in hepatic and pancreatic carcinogenesis

Previously, the HNF1A/MIA2 secretory axis has been demonstrated to have a tumor suppressor function in HCC^13^. However, we observed that both PDAC cancer cells widely expressed HNF1A and MIA2 and some cancers even expressed them at a very high level. This observation is in accordance with the data derived from a recent genome characterization, which revealed that MIA2 locates at a genomic region that is amplified in some PDAC cells^39^. It has also been shown that HNF1A enhances the expression of fibroblast growth factor receptor 4 (FGFR4) in pancreatic cancer cell lines - a growth factor receptor, which is over-expressed in PDAC^40^,^41^. These data do not support the notion that the HNF1A/MIA2 axis has a tumor suppressor function in pancreatic cancer (as compared to HCC) but rather argue for the opposite function. Indeed, Hnf1a deficiency in mice also induced a paradoxical consequence in the pancreas and liver in terms of carcinogenesis because it impaired large-T-antigen-induced growth and oncogenesis in pancreatic β cells but promoted proliferation of hepatocytes^33^. Therefore, the exact function of the HNF1A/MIA2 axis under physiological or pathological circumstances seems to be largely organ- and/or context-dependent.

### Acquired resistance of pancreatic cancer to ER stress is traded off with a susceptibility to chemotherapy

Recently, many genes belonging to the ER stress/UPR system have been demonstrated to be relevant in a variety of tumor entities^42^,^43^. Albeit for unknown reasons, it is speculated that more than one third of the human proteins enter the secretory pathway at the ER^15^. Thus, genetic variations in cargo-recognizing proteins such as MIA2 and MIA3 are hypothesized to have large biological effects because they may alter cargo protein transport efficiency or even the types of cargo proteins. Thereby, the biological impact of these common variations could be amplified through the secreted cargo proteins. For example, BiP (also known as heat shock 70 kDa protein 5), which is a major ER chaperone, affects the metastatic potential of gastric cancer cells and high expression of BiP is associated with a poor prognosis^44^,^45^. Although the role of the ER stress/UPR system in acute pancreatitis has been well characterized, its exact function in pancreatic carcinogenesis remains undefined to date^46^. In this regard, BiP has recently been identified as a novel tumor marker for pancreatic cancer, which is in line with our observations that half of the PDAC tissues were strongly BiP-positive^47^. In addition, we demonstrated that pancreatic cancer cells expressed high levels of genes belonging to the ER stress/UPR system including proximal signal sensors (PERK, ATF6 and ERN1), the chaperone lectins (Calnexin) and the folding catalysts (PDI). Further studies revealed that the ER stress/UPR system actually interacts with the MIA2^I141M^ variant in determining susceptibility of cancer cells to gemcitabine treatment. At the first glance, it seems difficult to understand why pancreatic cancer cell lines with the MIA2^I141M^ variant are highly sensitive to gemcitabine treatment while patients carrying this variant tend to have a more aggressive cancer phenotype (without chemotherapy). However, our data imply that gemcitabine - which is a generally genotoxic substance - preferably eliminates pancreatic cancer cells that are genetically and biologically more aggressive. These findings are consistent with results from a recent study in which PDACs can be classified into three major subtypes: classical, quasi-mesenchymal and exocrine-like, according to their gene expression profiles^32^. Among these subtypes, the quasi-mesenchymal subtype is the most aggressive one because patients belonging to this subtype have the worst prognosis. Interestingly however, cell lines with this gene expression signature were highly sensitive towards gemcitabine treatment in vitro. In contrast, cell lines with signatures of the less aggressive, classical subtype, were rather resistant to gemcitabine. These data argue for a biological “trade-off” phenomenon as described in other biological systems^48^, at least in the late stages of PDAC. Here, acquired resistance to natural stress, i.e. ER stress, is traded off with the susceptibility to genotoxic stress (e.g. chemotherapy).

We thus provide the first molecular explanation for an association between the MIA2^I141M^ common variant, the prognosis of resected PDAC patients and response to adjuvant chemotherapy. These findings are of clinical significance because they hold the promise to stratify resected PDAC patients into groups that are more likely to respond to systemic treatment. This would be of particular importance because the effect of adjuvant chemotherapy is rather modest and seems to be restricted to relatively few patients (e.g. patients with a high expression of human equilibrative nucleoside transporter 1 (hENT1))^49^,^50^. Stratification according to clinical scores (such as the McGill Brisbane Score^51^, ) in combination with genetic testing would ensure that patients with a low probability of responding to chemotherapy are spared a potentially toxic treatment.

## Methods

### Human material

The use of human material for this study was approved by the institutional review board of the Technische Universitaet Muenchen, Munich, Germany. All following methods/experimental protocols were carried out in accordance with the approved guidelines of the Ethics committee of the Technische Universitaet Muenchen, Munich, Germany. We obtained chronic pancreatitis (CP), pancreatic neuroendocrine tumor (PNT), PDAC, esophageal and colorectal tissues from patients on whom surgical resections were carried out. Written informed consent was obtained from the patients prior to the operation. Normal human pancreatic tissue samples were obtained through an organ donor program from previously healthy individuals, whenever no suitable recipient was found for the organ. The diagnoses of all samples were confirmed histologically. Samples were either snap-frozen in liquid nitrogen or were fixed in paraformaldehyde solution for 24 hours and were then paraffin-embedded for histological analysis. Genomic DNA used for genotyping analysis was isolated from paraffin-embedded tissues (cancer or normal tissues). Resections on PDAC patients have been performed between 2007 and 2010. Detailed clinical and pathological data (see Supplementary Table 2) were obtained from each patient and follow-ups amounted to at least 12 months. Patients with a survival of less than 2 months after surgery were excluded from the survival analysis in order to rule out surgery-related mortality. Genotyping was performed by B.K. and W.W.; both were blinded to the patient's outcomes at the time of analysis. Detailed information of other associated materials are available in the supplementary section.

### Immunohistochemistry analysis

Immunohistochemistry was performed using the Dako Envision System (Dako Cytomation GmbH, Hamburg, Germany). Consecutive paraffin-embedded tissue sections (3–5 mm thick) were deparaffinized and rehydrated using routine methods. Antigen retrieval was performed by pretreat ment of the slides in citrate buffer (pH 6.0; 10 mM Citric Acid, 0.05% Tween 20) in a microwave oven for 10 minutes. Endogenous peroxidase activity was quenched by incubation in deionized water containing 3% hydrogen peroxide at room temperature for 10 minutes. After blocking of nonspecific reactivity with TBS (pH 7.4; 0.1 M Tris Base, 1.4 M NaCl) containing 3% BSA or goat serum, sections were incubated with the respective antibody at 4°C overnight followed by incubation with horseradish peroxidase-linked goat anti-rabbit or mouse antibodies, followed by a color-reaction with diaminobenzidine and counterstaining with Mayer's hematoxylin.

### Cell culture

Human cell lines were cultured in 10 cm dishes either in DMEM or RPMI-1640 cell culture medium supplemented with 10% fetal bovine serum (FBS), 100 u/ml penicillin and 100 μg/ml streptomycin at 37°, 5% CO_2_.

### MRNA and cDNA preparation

All reagents for RNA extraction and the cDNA transcription kits were from Qiagen (Hilden, Germany) and were used according to the manufacturer's instructions.

### Quantitative Real-Time Polymerase Chain Reaction

Quantitative real time PCR (QRT-PCR) was carried out using the LightCyclerTM480 system with the SYBR Green 1 Master kit (Roche diagnostics, Penzberg, Germany). Expression of the target gene was normalized to the human housekeeping genes ACTB (β-actin) and HPRT1 (Hypoxanthine phosphoribosyltransferase 1) using the LightCyclerTM480 software release 1.5, version 1.05.0.39.

### Immunoblot Analysis

In brief, 20–80 μg of total cell lysate was loaded onto a 10% polyacrylamide gel and was then electrophoretically transferred to a nitrocellulose membrane. The membrane was blocked with 20 ml of Tween-20 (0.05%)-TBS (pH 7.4; 0.1 M Tris Base, 1.4 M NaCl) containing 3% or 5% milk for 1 h, followed by incubation with respective primary antibody overnight at 4°C. Membranes were washed 3 times with Tween-20 (0.05%)-TBS and were incubated with a horseradish peroxidase (HRP)-conjugated anti-mouse or anti-rabbit anti body (1:3000) for 1 h at room temperature. Signals were detected using the enhanced chemiluminescence system (ECL, Amersham Life Science Ltd., Bucks, UK). Films were scanned with a CanoScan 9900F scanner (Canon, Japan). Densitometric analysis was performed using the ImageJ software (http://imagej.nih.gov/ij).

### MIA2 ELISA

Human pancreatic cancer cell lines were cultured with 1 ml of medium in 6-well plates for 96 hours and supernatants were collected for further use. The MIA2 concentration in the supernatants was measured using a commercially available kit (Human MIA2 ELISA Development Kit, 900-K357, PEPROTECH, Hamburg, Germany) according to the manufacturer's instructions.

### High-resolution melting curve analysis

Reagents and material for the high-resolution melting curve analysis (HRM) were obtained from Roche Applied Science (LightCycler 480 High Resolution Melting Master, Roche diagnostics, Penzberg, Germany). Data were analyzed using the LightCyclerTM480 software release 1.5, version 1.05.0.39.

### Chemotherapy assay

Determination of the growth inhibition rates of each dose of the used drugs (depicted as growth fold of control) was carried out as described at http://www.dtp.nci.nih.gov/branches/btb/ivclsp.html. After incubation of the respective cells with different concentrations of gemcitabine (see below), cell growth was assessed by the 3-(4,5-dimethylthiazole-2-yl)2,5-diphenyltetrazolium bromide (MTT; 5 mg/ml in PBS; Sigma Aldrich, St. Louis, MO) colorimeric growth assay. Growth inhibition is calculated as: (Ti-Tz)/(C-Tz) [time zero, (Tz), growth of control, (C), and cell growth in the presence of the drug at the different concentration levels (Ti)]. The introduction of Tz in the formula serves to reduce the experimental variations resulting from different initial numbers of seeded cells, thus facilitating interpretation of the data. Gemcitabine was added 24 hr after seeding of the cells at increasing concentrations (10, 50, 100, 500, and 1,000 nM). 0.01% PBS was used as a control. After 48 hours, an MTT test was performed to assess cell viability. All experiments were repeated three times.

### Site-directed mutagenesis

The site-directed mutagenesis was performed using the “QuikChange Lightning Multi Site-Directed Mutagenesis Kit” purchased from Stratagene (Stratagene, Agilent Technologies, CA, USA) according to the manufacturer's instructions. Briefly, the pCMV6-Entry vector containing the full-length human MIA2 was used as the backbone to generate the respective MIA2 variant-expressing vectors. Newly generated vectors were confirmed by sequencing before transfection.

### Transfection and generation of stable MIA2 expressing pancreatic cancer cell lines

Su86.86 cells were transfected with different MIA2 expressing vectors using the Lipofectamine 2000 transfection reagent (Invitrogen, Carlsbad, California) according to the manufacturer's instructions. For transient expression of HNF1A, the expression vector from Origene was used (RC211201, Origene, Suite 200 Rockville, MD). For generation of stable clones, Su86.86 cells were transfected with 20 μg of each MIA2 expressing vector or empty vector (as a control) in a 10 cm dish and selection medium containing G418 (0.5 mg/ml) was applied for 24 hours after transfection. After culturing with selection medium for 7 days, cells were reseeded into 96-well plates at limited dilutions (50, 10, 5, 1 and 0.5 cells per well). 3 days after plating, wells containing a single clone were marked and its clones were further expanded and screened for MIA2 expression by QRT-PCR assays and by immuno-blot analysis. The clones expressing the MIA2 variants at a similar level were sequenced to confirm the genotype of exogenous MIA2 before functional characterization.

### Real-Time PCR arrays

Reagents and material for the Real-Time PCR array were obtained from SABiosciences (SABiosciences Corporation, Frederick, USA). The assay was performed according to the manufacturer's instruction. Human Unfolded Protein Response (PAHS-089F) and Human Extracellular Matrix and Adhesion Molecules (PAHS-013F) arrays were used. In total, 23 RNA samples of PDAC tissues were analyzed (one array per cancer tissue). Data were analyzed using the web-based software from SABiosciences (www.sabiosciences.com/pcr/arrayanalysis.php). Group-wise comparisons were performed using the Student's t-test.

### Statistical analysis

For statistical analyses, either the GraphPad Prism 5 Software (GraphPad, San Diego, CA, USA) or the IBM SPSS 19 Software (IBM Corp., Armonk, NY) was used. The Chi-square test was used to compare the distribution of categorical variables among PDAC patients with and without the MIA2^I141M^ variant. The Log-rank (Mantel-Cox) Test was used to compare the survival of patients with and without the MIA2^I141M^ variant. Unless otherwise stated, an unpaired t-test was used for group-wise comparisons. The level of statistical significance was set at p < 0.05. Results are expressed as mean ± standard deviation (SD) unless indicated otherwise.

## Author Contributions

B.K. designed and conducted the experiments, interpreted the data and wrote the paper. W.W.W., N.V. and X.H. performed experiments. C.J. acquired clinical data of the pancreatic cancer patients and provided statistical expertise. U.N. acquired clinical data and provided genomic DNA of colon cancer patients. H.F., I.E. and M.E. contributed to the discussion and helped with the writing of the paper. J.K. and C.W.M. designed the study, interpreted the data, wrote the manuscript and supervised the project.

## Supplementary Material

Supplementary Information Supplementary Information
