# Supplementary material for: A common genetic variation of melanoma inhibitory activity-2 labels a subtype of pancreatic adenocarcinoma with high endoplasmic reticulum stress levels
Source: Sci Rep. 2015 Feb 6;5:8109. doi: 10.1038/srep08109 (PMC4319175; doi:10.1038/srep08109)

Supplementary section to:

A common genetic variation of melanoma inhibitory activity-2 labels a subtype of pancreatic adenocarcinoma with high endoplasmic reticulum stress levels

Bo Kong[1], Weiwei Wu[1], Nataliya Valkovska[1], Carsten Jäger[1], Xin Hong[1], Ulrich Nitsche[1], Helmut Friess[1], Irene Esposito[2], Mert Erkan[1], Jörg Kleeff[1,*] and Christoph W. Michalski[1,3,*]

[1] Department of Surgery, Technische Universität München, Munich, Germany

[2] Institute of Pathology, Technische Universität München, Munich, Germany

[3] Department of Surgery, University of Heidelberg, Germany

[*] equal contribution

Supplementary Figure Legends.

Figure 1.

(**a**) Immunoblot analysis demonstrates successful transient expression of HNF1B in Panc1 cells; loading control: GAPDH. (**b**) Transient expression of HNF1B has no effect on the expression of MIA2 mRNA levels; Control: non-transfected Panc1 cells, HNF1B: HNF1B-transfected Panc1 cells, HPRT1: housekeeping gene. One of three independent experiments is shown.

Figure 2.

(**a**) HRM analysis of $MIA2^{WT}$ and the $MIA2^{I141M}$ variants in genomic DNA of PDAC patients demonstrates highly different curve patterns, 617AA: wild type, 617AG: heterozygous, 617GG: homozygous. (**b**) Randomly selected samples were sequenced to confirm the results. (**C**) HRM

analysis of MIA2^{WT} and the MIA2^{D547H} variant in genomic DNA of PDAC patients demonstrates highly different curve patterns, 1833GG: wild type, 1833GC: heterozygous, 1833CC: homozygous.

Figure 3.

CDNAs from Su^{WT}, Su^{I141M}, Su^{D547H} and Su$^{I141M\&D547H}$ were sequenced to confirm the introduced polymorphisms.

Figure 4.

(**a**) Among the three UPR arms, the ERN1/XBP1 arm and a number of downstream molecules (DNAJC4, HERPUD1, PPIA and HSPA1L) are increased more than 50% in MIA2^{I141M} variant carries. Two of three ECM and adhesion molecules are decreased (LAMA1 and MMP12) and one (ITGA2) is up-regulated. (**b**) A semi-quantitative RT-PCR confirms increased expression of unspliced (u-)XBP1 in the MIA2^{I141M} variant carrier tumor samples. Expression of spliced (s-)XBP1 is unchanged. (**C**) There is no difference in the expression of s-XBP1 by QRT-PCR. Data are presented as relative expression (normalized to the median expression of s-XBP1 in WT samples); n.s.: not significant.

Figure 5.

35% (21/60) and 48% (32/67) of the PDAC samples are immune-positive for the UPR sensors ATF6 and PERK, respectively. 63% (39/67) and 46% (31/68) of the PDAC sections are positive for the target molecules of the UPR, PDI and Calnexin, respectively (scale bar: 100 µm).

Supplementary Tables.

Supplementary Table 1

Frequency of the MIA2^{I141M} variant in organ donors and different disease entities

Samples	rs11845046 (MIA2^{I141M})				Total
	617 AA	617 AG	617 GG	Minor allele frequency (MAF)	

Donor pancreas	13	13	0	0.50	26
CP	13	5	0	0.28	18
PNT	22	12	4	0.42	38
PDAC	180	80	17	0.35	277
Colorectal cancer	147	71	12	0.36	230
Esophageal cancer	26	10	3	0.33	39
In total	401	191	36	0.36	628

CP: chronic pancreatitis, PNT: pancreatic neuroendocrine tumor

Supplementary Table 2

Clinical characteristics of the PDAC patient cohort

	$MIA2^{WT}$	$MIA2^{I141M}$	p-value	Total
Age (years, median)	66	66	0.92[1]	-
Gender				
Male	32	19	0.43[2]	51
Female	27	21		48
Tumor size (T)				
T1-T2	4	5	0.33[2]	9
T3-T4	55	35		90
Lymph node status (N)				
N0	17	16	0.25[2]	33
N1	42	24		66
Metastasis (M)				
M0	53	36	0.98[2]	89
M1	6	4		10
Grading (G)				
G1	4	3	0.85[2]	7
G2	27	16		43
G3	28	21		49

R status				
R0	31	19	0.87[2]	50
R1/R2	20	15		35
unknown	8	6		14
Chemotherapy				
Yes	46	34	0.38[2]	80
No	13	6		19

[1]: Mann-U-Whitney-Test; [2]: Chi^2-test

Supplementary Table 3

Differentially expressed genes related to the UPR in PDACs with the $MIA2^{I141M}$ variant or the $MIA2^{WT}$

	**Symbol**	**Description**	**Fold Change**	**P value[1]**	**Function**
NM_001675	ATF4	Activating transcription factor 4 (tax-responsive enhancer element B67)	1.3	0.012	Transcription Factor
NM_007348	ATF6	Activating transcription factor 6	1.3	0.018	Transcription Factor
NM_018981	DNAJC10	DnaJ (Hsp40) homolog, subfamily C, member 10	1,4	0.018	Unfolded Protein Binding, Protein Folding, Protein Disulfide Isomerization, Heat Shock Proteins
NM_006260	DNAJC3	DnaJ (Hsp40) homolog, subfamily C, member 3	1.2	0.042	Heat Shock Proteins
NM_005528	DNAJC4	DnaJ (Hsp40) homolog, subfamily C, member 4	1.5	0.014	Unfolded Protein Binding, Protein Folding, Heat Shock Protein
NM_014674	EDEM1	ER degradation enhancer, mannosidase	1.2	0.013	ER Protein Folding Quality Control,

		alpha-like 1			ERAD
NM_025191	EDEM3	ER degradation enhancer, mannosidase alpha-like 3	1.4	0.046	ER Protein Folding Quality Control, Ubiquitination
NM_032025	eIF2A	Eukaryotic translation initiation factor 2A, 65kDa	1.3	0.013	Regulation of Translation
NM_001433	ERN1	Endoplasmic reticulum to nucleus signaling 1	1.5	0.044	Transcription Factor, Apoptosis
NM_014685	HERPUD1	Homocysteine-inducible, endoplasmic reticulum stress-inducible, ubiquitin-like domain member 1	1.5	0.019	ERAD, Ubiquitination
NM_005527	HSPA1L	Heat shock 70kDa protein 1-like	1.5	0.004	Heat Shock Protein
NM_003791	MBTPS1	Membrane-bound transcription factor peptidase, site 1	1.3	0.043	Regulation of Cholesterol Metabolism, ERAD, Transcription Factor
NM_021130	PPIA	Peptidylprolyl isomerase A (cyclophilin A)	1.5	0.000	Unfolded Protein Binding, Protein Folding
NM_003262	SEC62	SEC62 homolog (S. cerevisiae)	1.3	0.022	ERAD, Ubiquitination
NM_005080	XBP1	X-box binding protein 1	1.6	0.041	Transcription Factor

ERAD: ER Associated Degradation; [1]: P values were generated using the following on-line array analysis tool: (http://pcrdataanalysis.sabiosciences.com/pcr/arrayanalysis.php)

Supplementary Table 4

Differentially expressed genes related to ECM and adhesion in PDAC tissues with the MIA2^{I141M} variant or the MIA2^{WT}

	Symbol	Description	Fold Change	P value[1]	Function
NM_002203	ITGA2	Integrin, alpha 2 (CD49B, alpha 2 subunit of VLA-2 receptor)	1.9	0.045	Cell-Matrix Adhesion, Transmembrane Molecule
NM_005559	LAMA1	Laminin, alpha 1	0.4	0.008	Basement Membrane Constituent, Adhesion Molecule
NM_000201	ICAM1	Intercellular adhesion molecule 1	0.7	0.038	Transmembrane Molecule, Cell-Cell Adhesion
NM_002426	MMP12	Matrix metallopeptidase 12 (macrophage elastase)	0.4	0.045	ECM Protease

[1]: P values were generated using the following on-line array analysis tool: (http://pcrdataanalysis.sabiosciences.com/pcr/arrayanalysis.php)

Supplementary Materials and Methods

Human cell lines

HEK293 cells (a kind gift from PD Dr. Klaus-Peter Janssen, Molecular Tumor Biology, Department of Surgery, Technische Universitaet Muenchen) and seven human pancreatic cancer cell lines – Aspc-1, Bxpc-3, Colo-357, MiaPaCa-2, Su86.86, Panc-1 and T3M4 (obtained from ATCC, Wesel, Germany) – were used for the experiments. The human pancreatic cancer cell lines used for functional analyses were tested for their integrity by the authentication service of the “DSMZ” (German Collection of Microorganisms and Cell Cultures, Braunschweig, Germany).

Plasmids, siRNAs and primers

The human MIA2 expression vector (RC224284), the HNF1A expression vector (RC211201) and the HNF1B expression vector (SC122562) were purchased from Origene (VWR International GmbH, Darmstadt, Germany). Human MIA2 siRNA and ERN1 siRNAs were purchased from Ambion Applied Biosystems (Carlsbad, California, USA) and QIAGEN (Hilden, Germany), respectively. All primers used in this study were synthesized by Metabion (Martinsried, Germany).

Sequences of siRNA used for gene-silencing experiments:

Name	Sense (5'→3')	Antisense (5'→3')
MIA2-siRNA1#	GGAGUAGAAAAAUAGCAGUTT	ACUGCUAUUUUUCUACUCCGA
MIA2-siRNA2#	CAGACGAAUCUGAUACUAUTT	AUAGUAUCAGAUUCGUCUGTT
ERN1-siRNA1#	GGACGUGAGCGACAGAAUATT	UAUUCUGUCGCUCACGUCCTG
ERN1-siRNA2#	GCACGGACGUCAAGUUUGATT	UCAAACUUGACGUCCGUGCTG

Sequences of primers used for QRT- or RT-PCR analysis of human genes:

Gene Name	Sense (5'→3')	Antisense (5'→3')
MIA2	ATGGCAAAATTTGGCGTTC	CCTGCCCACAAATCTTCC
ERN1	CGAGGAGGTGGGGGAAGCGA	GCTGCCGGGTGTTGGGGAAA
XBP1-unspliced	ATGGCTTTGGGCAGTGGCTGG	GGGGATGGATGCCCTGGTTGC
XBP1-spliced	CCTGCACCTGCTGCGGACTCA	GAGTTAAGACAGCGCTTGGG
XBP1	GGTAAGGAACTGGGTCCTT	AGAGAGGCGGGAGAGCCGTG
HNF1B	CCTCCAAAGCCCACGGCCTG	AGCCGTGGGAGAGCAGAGGG
ACTB	CTACGTCGCCCTGGACTTCGAGC	GATGGAGCCGCCGATCCACACGG
HPRT1	GCTTTCCTTGGTCAGGCAGTATAAT	AAGGGCATATCCTACAACAAACTG

Sequences of primers used for mutagenesis for generating the MIA2 variants:

Name	Sense (5'→3')
MIA2-A617G	GTGAATTAAACGGTGATTATGGTGAAAATATGTATCCTTATGAAGAAGATAAAG
MIA2-G1833C	ATTTTGAACCCTCATCTTCTAAACATAGTGATGAAAATTCGAACCC

Sequences of primers used for sequencing of the human MIA2 cDNA:

Position of *MIA2* cDNA	Sequence (5'→3')
Sense-134	CTCTACAACCTGAACAATTGGCTTAA
Sense-751	TATCGGAAGTACCAGTGAATCAAAAG
Sense-1329	ATTTTGGTTTTGCTATACTAGGCTTT
Sense-1237	TAAAGAAGCCACAGTTCCATGTACAG
Sense-1731	GATATTCAAAAGTTCATACAGTCTG
Antisense-822	GGAATACGATCCTGTTCCATACTTTC
Antisense-1354	AAAGCCTAGTATAGCAAAACCAAAAT
Antisense-1559	ATGGTATAGTATCAGATTCGTCTGTT
Antisense-1769	AGACCATATCTGACAGACTGTATGAA
Antisense-1857	GATGGTTTCGAATTTTCATCACTATC
Antisense-1959	TGAGAAGACAAAGAGTTATCAGTTGA
Antisense-2247	TGACAAAACATTCATATAAAGAGAAA

Sequences of primers used for high-resolution melting curve analysis of the MIA2 variants:

Variant name	Sense (5'→3')	Antisense (5'→3')

MIA2^{I141M}	AGTGAATTAAACGGTGATTATGGTG	CCTCTAATGCTGGAACTTGGTCT
MIA2^{D547H}	AGAGTTACCTACGAGAATTCACGAA	CTATTTAGCAGGGTATTTTCCACG

Antibodies

Antibody name	Catalog Number	Application	Company
Rabbit Anti-MIA2 pAb	500-P255	WB, IHC	PeproTech<br>(Hamburg, Germany)
Rabbit Anti-HNF1A pAb	NBP1-33596	WB, IHC	Novus Biologicals<br>(Cambridge, UK)
Rabbit Anti-ERN1 pAb	HPA027730	IHC	Sigma-Aldrich<br>(Munich, Germany)
Rabbit Anti-ERN1 mAb	3294	WB	Cell Signaling Technology<br>(NEB, Frankfurt/Main, Germany)
Rabbit Anti-ATF6 pAb	NBP1-41439	WB, IHC	Novus Biologicals
Rabbit Anti-BiP mAb	3177	WB, IHC	Cell Signaling Technology
Rabbit Anti-PDI mAb	3501	WB, IHC	Cell Signaling Technology
Rabbit Anti-Calnexin mAb	2679	IHC	Cell Signaling Technology
Rabbit Anti-PERK mAb	5683	WB, IHC	Cell Signaling Technology

WB: Western-blot; IHC: Immunohistochemistry

Supplementary Figure 1

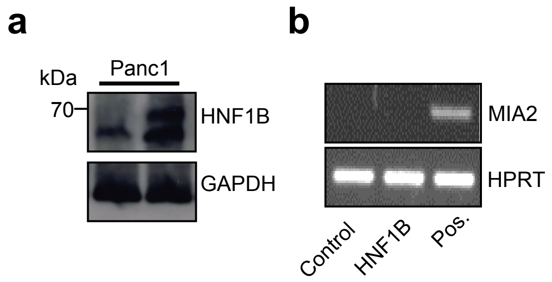

Supplementary Figure 2

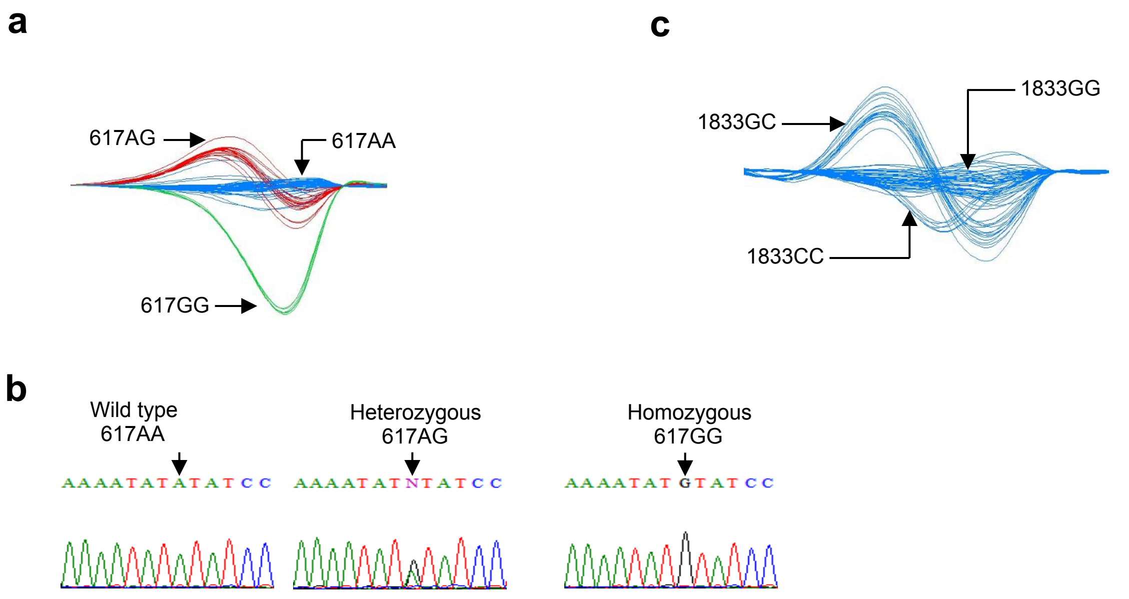

Supplementary Figure 3

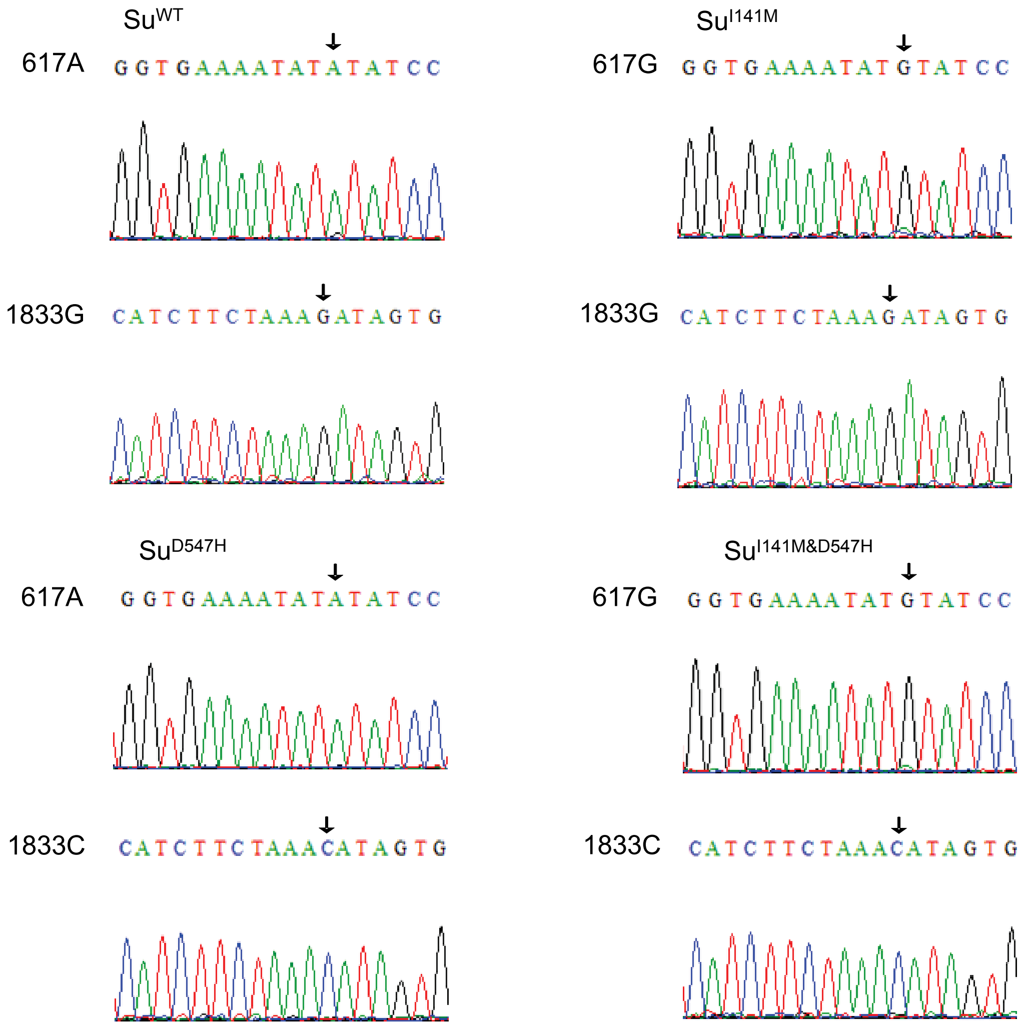

Supplementary Figure 4

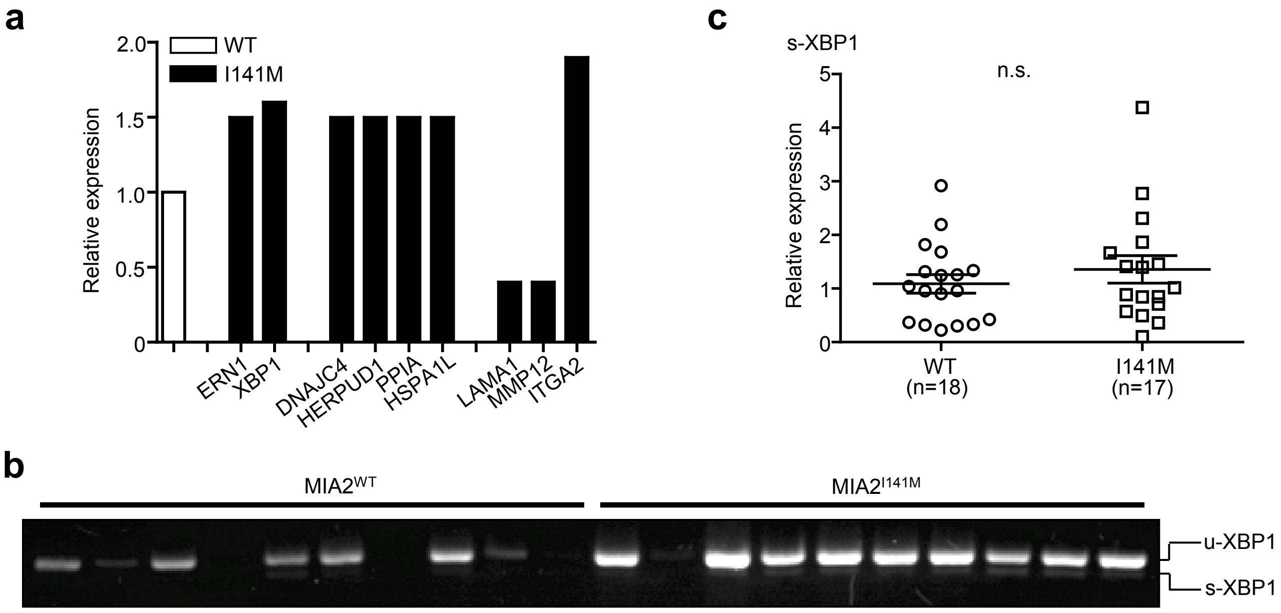

Supplementary Figure 5

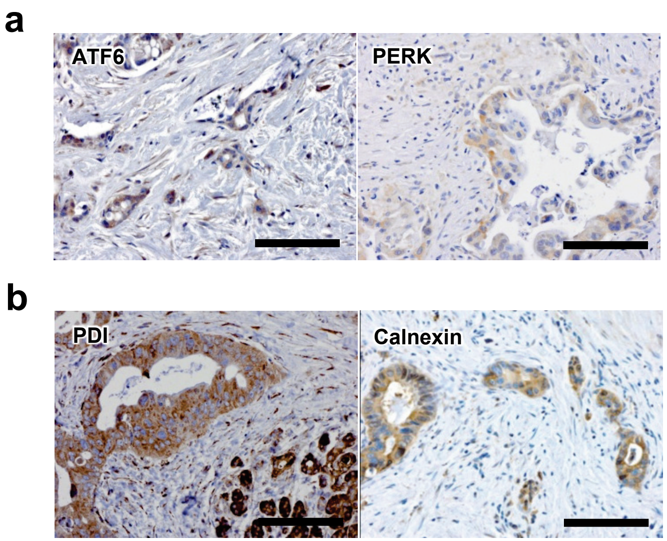

Uncropped blots/images on the following pages.

Figure 1c

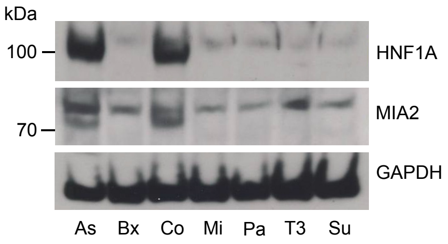

HNF1A

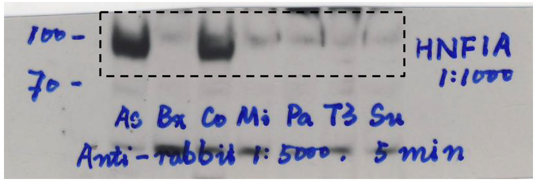

MIA2

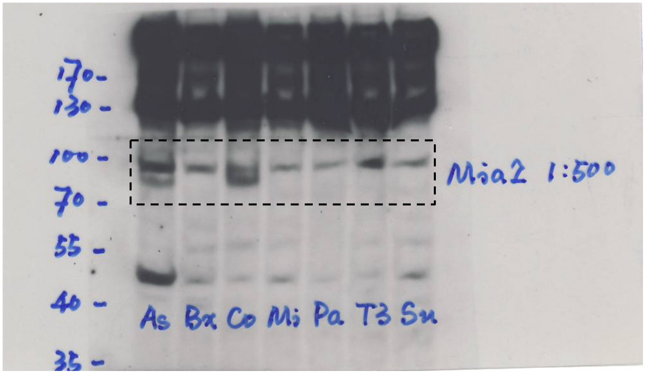

GAPDH

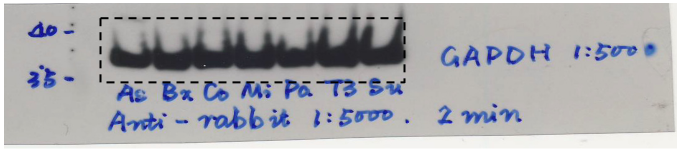

Figure 1e

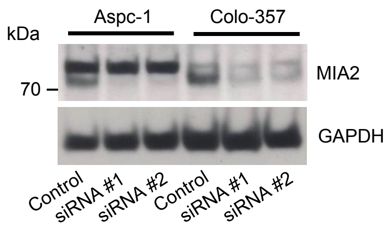

MIA2

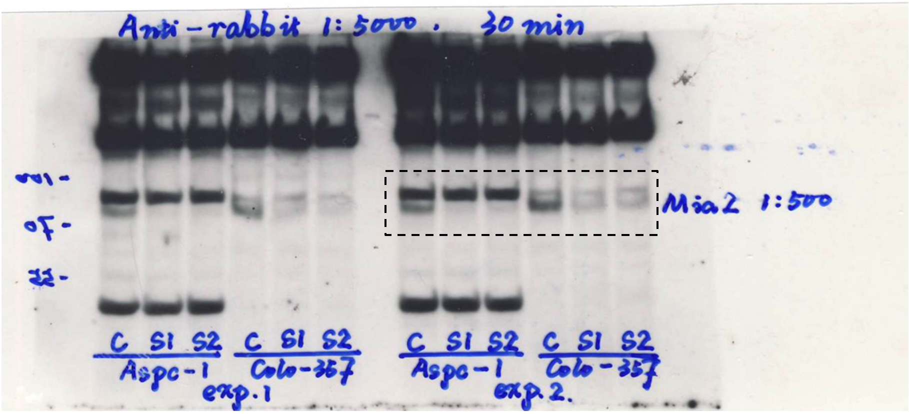

GAPDH

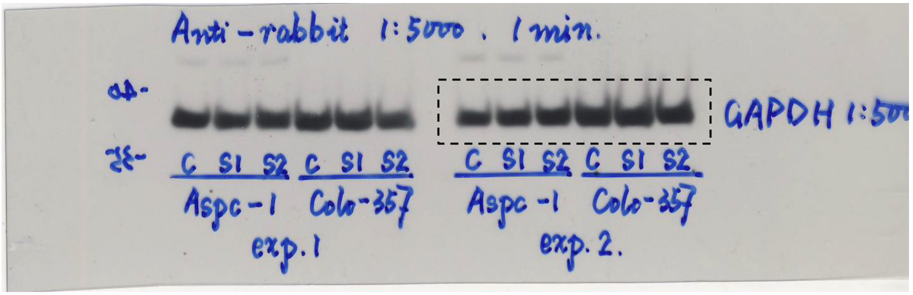

Figure 1f

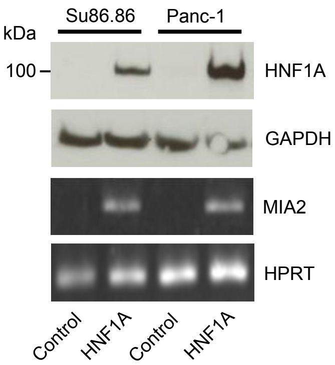

HNF1A

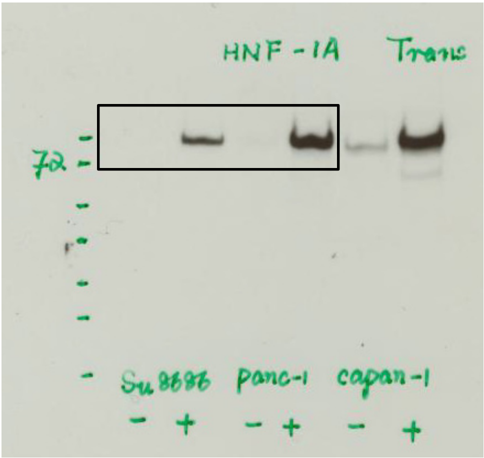

GAPDH

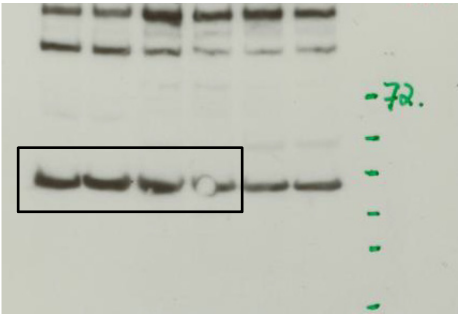

HPRT

MIA2

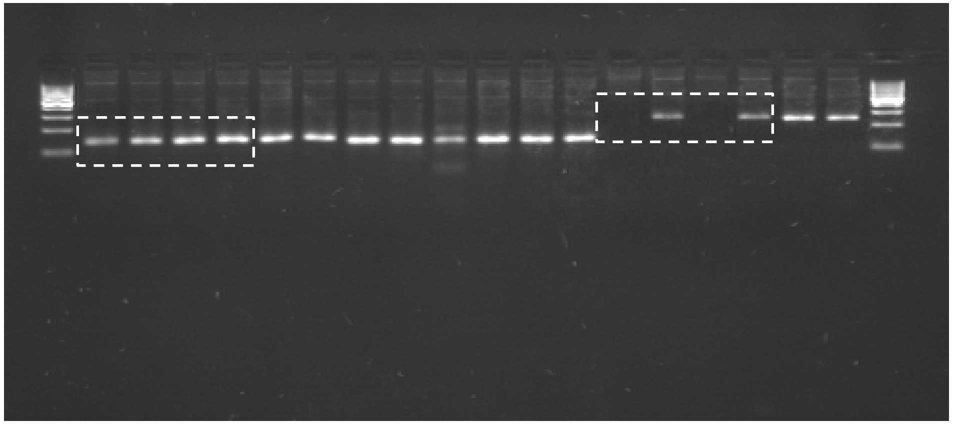

Figure 3b

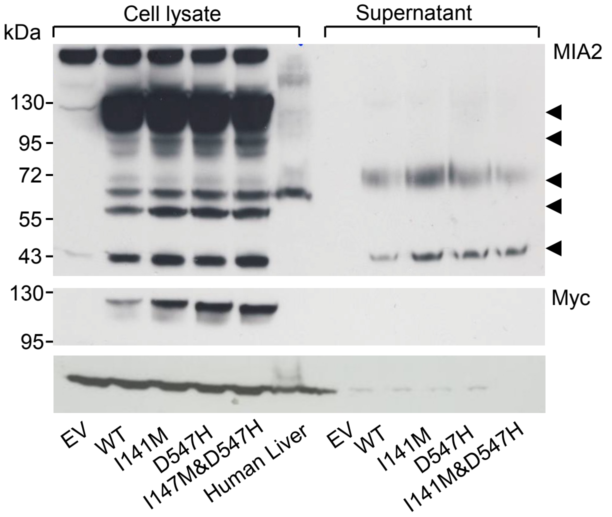

MIA2

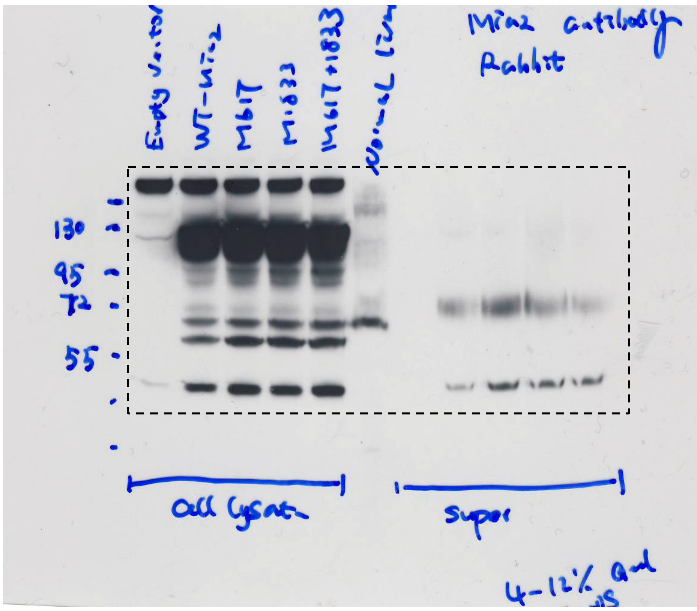

Myc

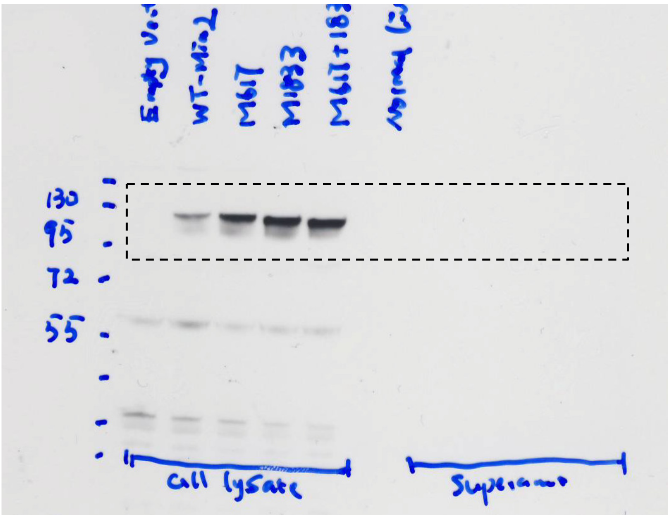

GAPDH

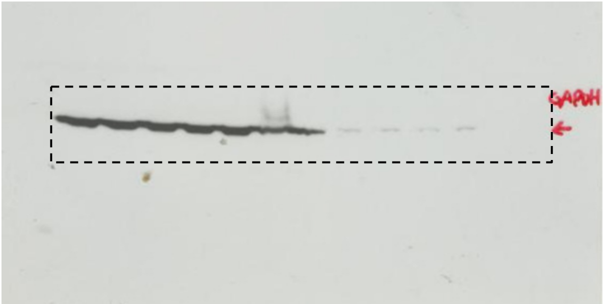

Figure 3c

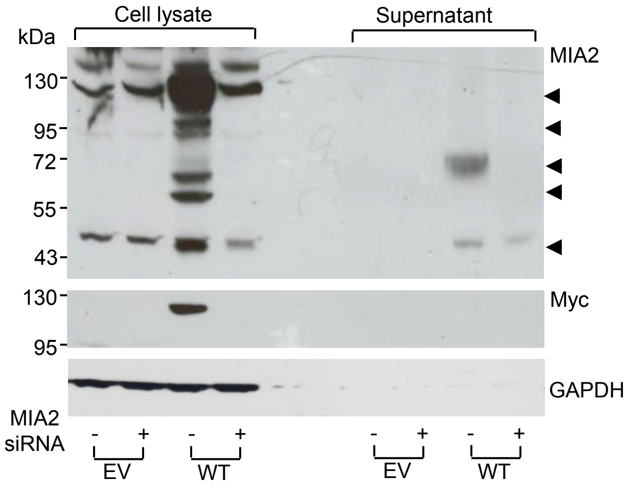

MIA2

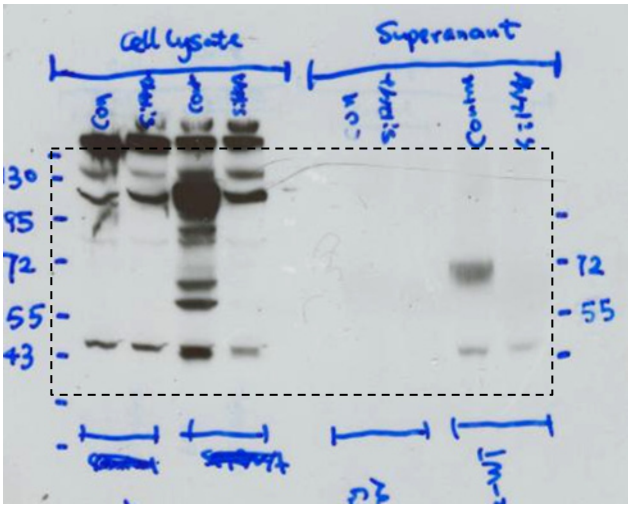

Myc

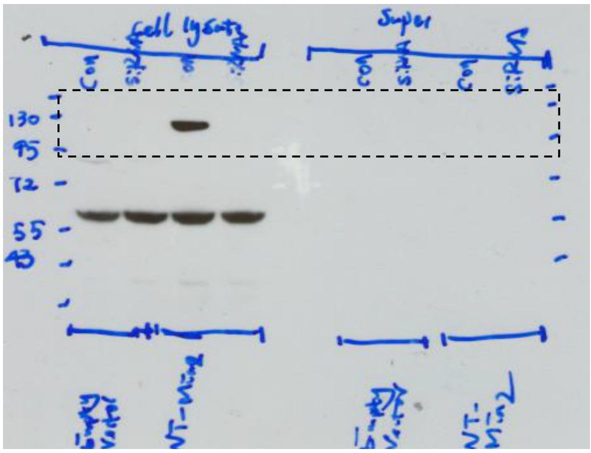

GAPDH

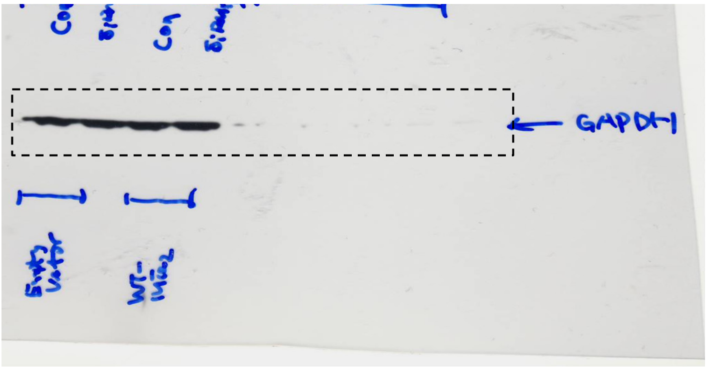

Figure 3d

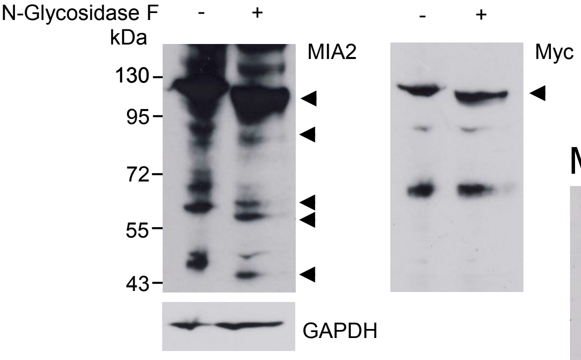

MIA2

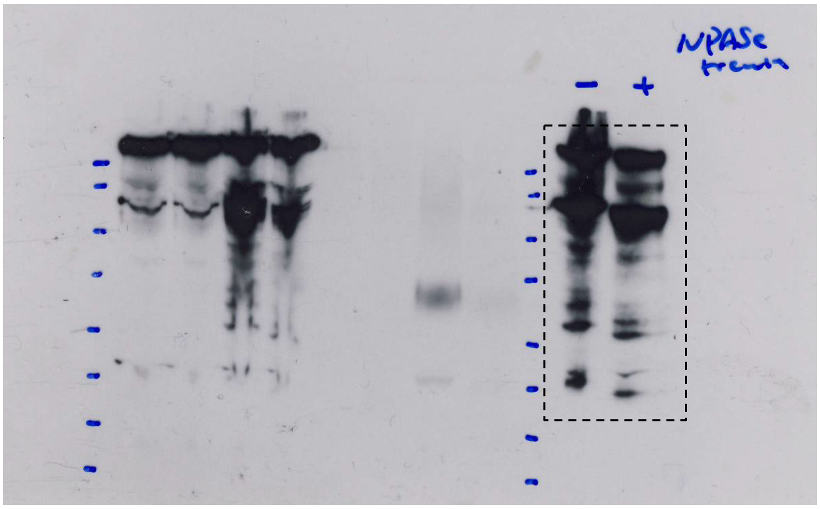

Myc

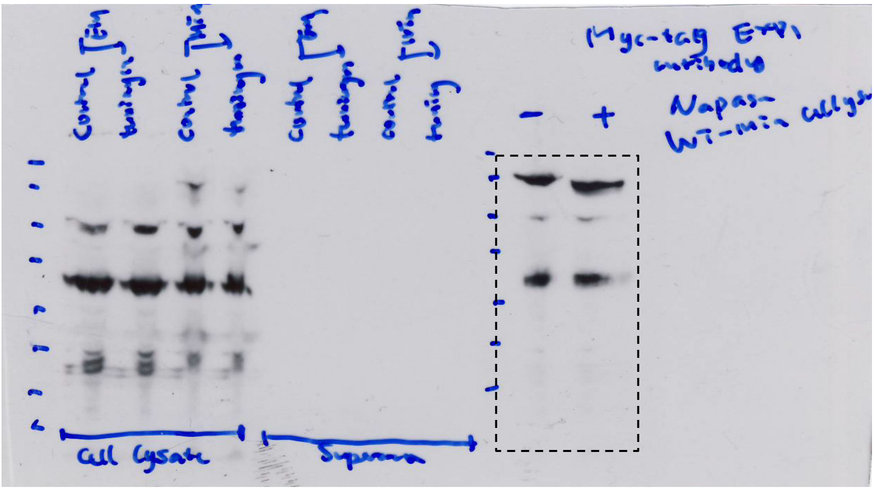

GAPDH

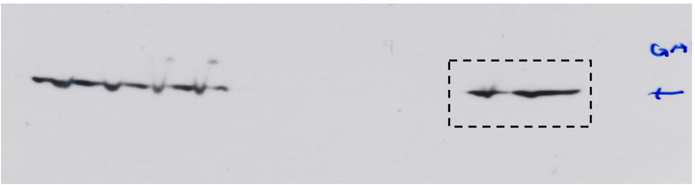

Figure 3e

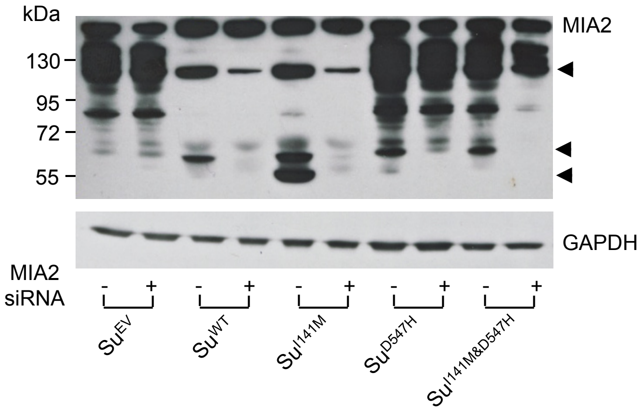

MIA2

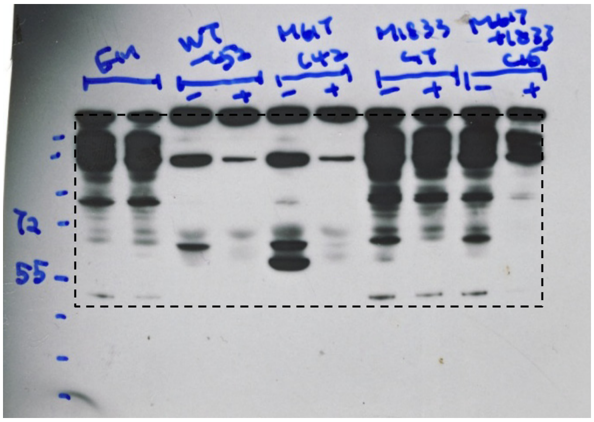

GAPDH

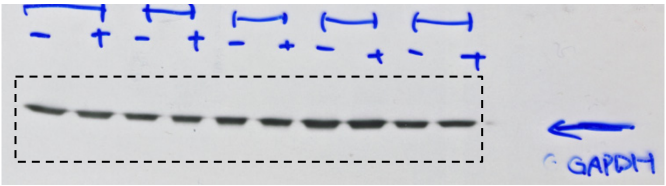

Figure 4e

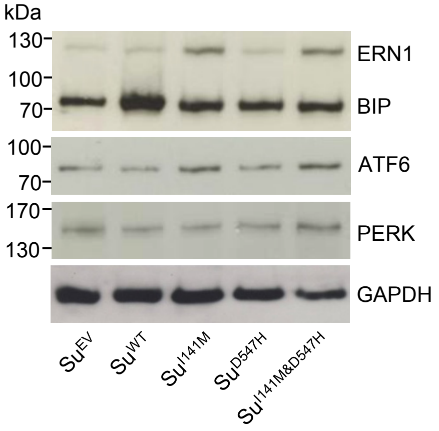

ERN1 + BiP

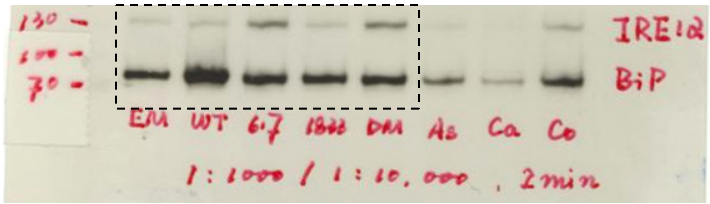

ATF6

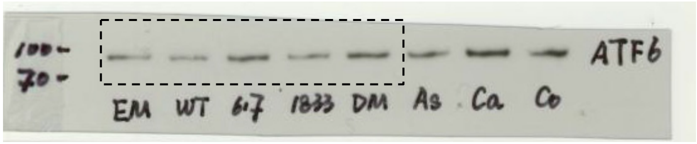

PERK

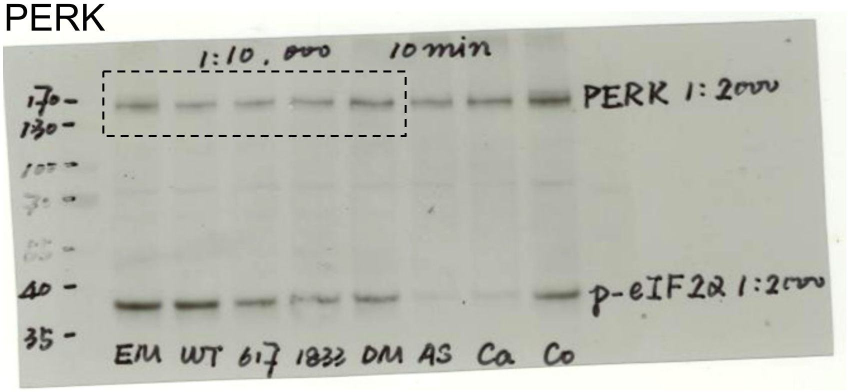

GAPDH

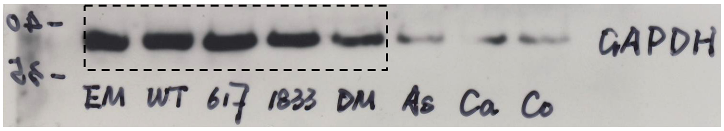

Figure 4f

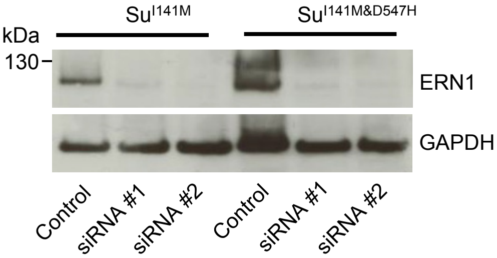

ERN1

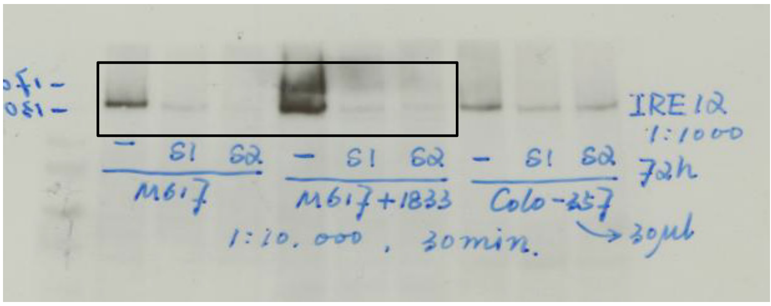

GAPDH

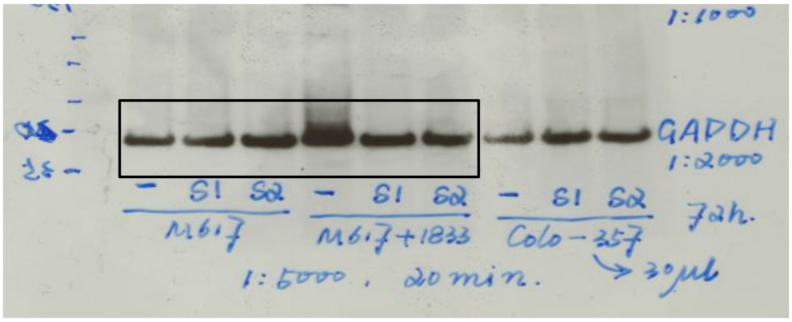

Supplementary Figure 1

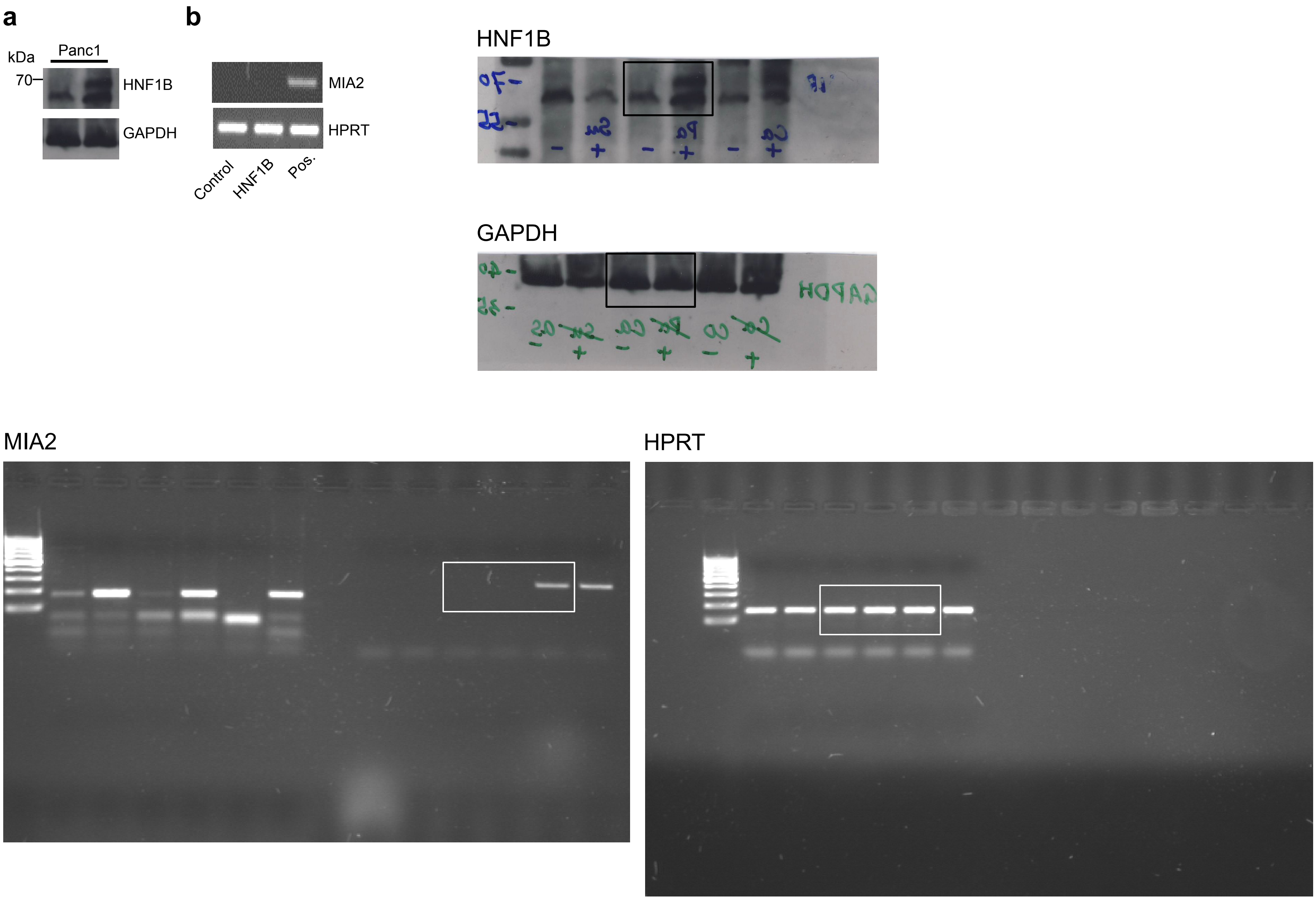

Supplementary Figure 4

b

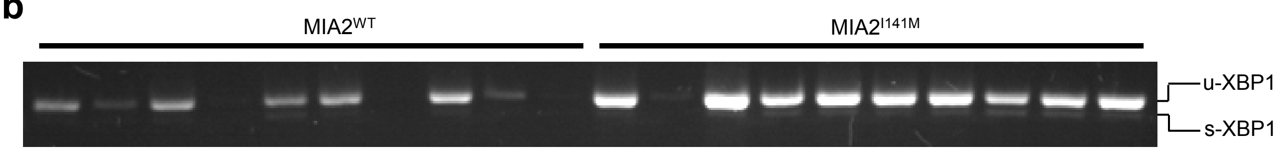

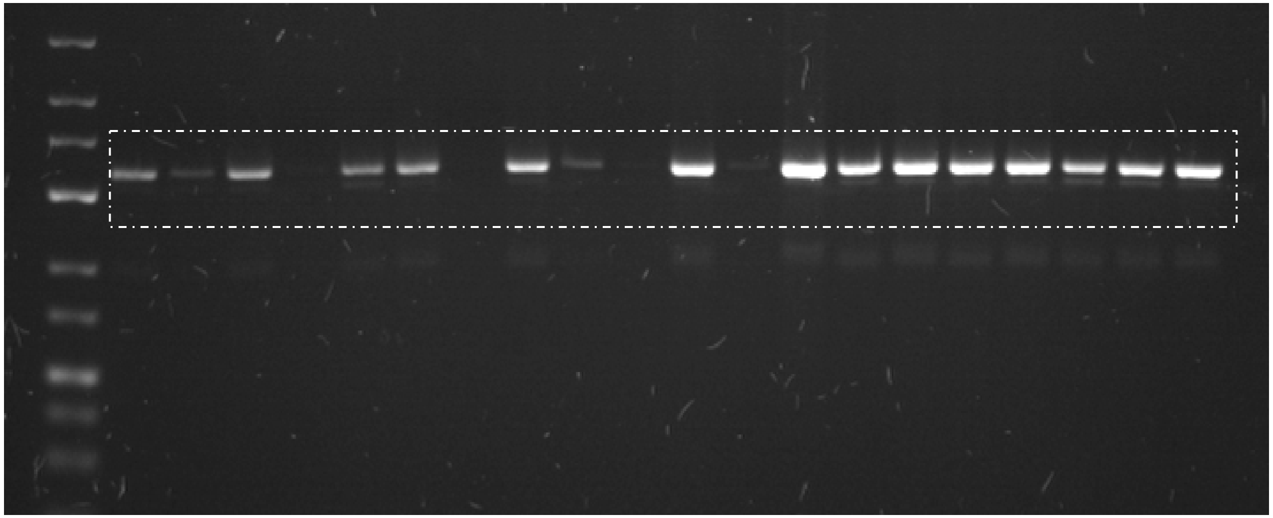

Supplement: Supplementary Information [file srep08109-s1.pdf]
